# Transcriptomic profiles of muscular dystrophy with myositis (*mdm*) in extensor digitorum longus, psoas, and soleus muscles from mice

**DOI:** 10.1186/s12864-022-08873-2

**Published:** 2022-09-17

**Authors:** Pabodha Hettige, Uzma Tahir, Kiisa C. Nishikawa, Matthew J. Gage

**Affiliations:** 1 Department of Chemistry, University of Massachusetts Lowell, Lowell, MA 01854 USA; 2 UMass Movement Center, University of Massachusetts Lowell, Lowell, MA 01854 USA; 3 Department of Biological Sciences, Northern Arizona University, Flagstaff, AZ 86011 USA

**Keywords:** RNA-Seq, *Mdm*, Mitochondria

## Abstract

**Background:**

Titinopathies are inherited muscular diseases triggered by genetic mutations in the titin gene. Muscular dystrophy with myositis (*mdm*) is one such disease caused by a LINE repeat insertion, leading to exon skipping and an 83-amino acid residue deletion in the N2A-PEVK region of mouse titin. This region has been implicated in a number of titin—titin ligand interactions, hence are important for myocyte signaling and health. Mice with this *mdm* mutation develop a severe and progressive muscle degeneration. The range of phenotypic differences observed in *mdm* mice shows that the deletion of this region induces a cascade of transcriptional changes extending to numerous signaling pathways affected by the titin filament. Previous research has focused on correlating phenotypic differences with muscle function in *mdm* mice. These studies have provided understanding of the downstream physiological effects resulting from the *mdm* mutation but only provide insights on processes that can be physiologically observed and measured. We used differential gene expression (DGE) to compare the transcriptomes of extensor digitorum longus (EDL), psoas and soleus muscles from wild-type and *mdm* mice to develop a deeper understand of these tissue-specific responses.

**Results:**

The overall expression pattern observed shows a well-differentiated transcriptional signature in *mdm* muscles compared to wild type. Muscle-specific clusters observed within the *mdm* transcriptome highlight the level of variability of each muscle to the deletion. Differential gene expression and weighted gene co-expression network analysis showed a strong directional response in oxidative respiration-associated mitochondrial genes, which aligns with the poor shivering and non-shivering thermogenesis previously observed. Sln, which is a marker associated with shivering and non-shivering thermogenesis, showed the strongest expression change in fast-fibered muscles. No drastic changes in MYH expression levels were reported, which indicated an absence of major fiber-type switching events. Overall expression shifts in MYH isoforms, MARPs, and extracellular matrix associated genes demonstrated the transcriptional complexity associated with *mdm* mutation. The expression alterations in mitochondrial respiration and metabolism related genes in the *mdm* muscle dominated over other transcriptomic changes, and likely account for the late stage cellular responses in the *mdm* muscles.

**Conclusions:**

We were able to demonstrate that the complex nature of *mdm* mutation extends beyond a simple rearrangement in titin gene. EDL, psoas and soleus exemplify unique response modes observed in skeletal muscles with *mdm* mutation. Our data also raises the possibility that failure to maintain proper energy homeostasis in *mdm* muscles may contribute to the pathogenesis of the degenerative phenotype in *mdm* mice. Understanding the full disease-causing molecular cascade is difficult using bulk RNA sequencing techniques due to intricate nature of the disease. The development of the *mdm* phenotype is temporally and spatially regulated, hence future studies should focus on single fiber level investigations.

**Supplementary Information:**

The online version contains supplementary material available at 10.1186/s12864-022-08873-2.

## Background

Titinopathies are inherited striated muscle-associated diseases linked to genetic mutations in the titin gene [1]. Mouse titin protein is encoded for by 347 exons (Ensembl transcript version ENSMUST00000099981.9, NCBI Accession: BN001114.1) and spans across a 278,567 bp long genomic region. The titin gene codes for the largest and the third most abundant protein expressed in striated muscles [2]. Due to its size and the number of potential alternative splice sites within the gene, there are potentially billions of titin isoforms expressed by this single gene [3]. While variability in isoforms allows healthy sarcomeres to fine-tune muscle function, mutations interrupting its native protein structure can lead to titin-associated diseases [4].

Muscular dystrophy with myositis (*mdm*) is one such titin-associated disease in mice, and caused by a complex rearrangement at the N2A-PEVK region of titin, that disrupts one of the most important active sites for titin-based force regulation [3, 4]. The disease is caused by a 779 bp genomic deletion and a 2.4-kb 5’-truncated LINE-1 retrotransposon insertion at the N2A-PEVK junction of titin, leading to an in-frame 248 nucleotide deletion in mature mRNA [5–7]. This mutation leads to 53 amino acid loss in immunoglobulin domain 83 (Ig83) at the end of the N2A domain and a loss of 30 amino acid at the beginning of PEVK. Even though the *mdm* deletion accounts for a small fraction (0.2%) of titin’s largest known isoform, the homozygous recessive mice show a severe and progressive muscle degeneration [1]. Fore and hind limb muscles are severely affected by the *mdm* deletion, and symptoms include kyphosis in the spine, rigid gait, smaller body mass, and premature death as early as ~ 2 months after birth [7]. Fiber size variation and abundance of central nuclei indicate repetitive cycles of muscle degeneration and regeneration [7]. Increased collagen content, passive tension [1, 8], and weak active force generation [9] are also characteristics of *mdm* deletion. Mice with the *mdm* deletion lose their ability to grow after weaning [7], suggesting impairments in hypertrophy signaling, an essential element for healthy muscle growth. Weaker shivering and non-shivering thermogenesis and muscle tremor is often observed in *mdm* mice [6, 10]. A majority of the observed molecular changes are unique to *mdm* muscles and distinct from typical alterations seen in other muscular dystrophies [1].

The *mdm* mutation interrupts the calpain3 (Capn3) binding site at the N2A signalosome, but attenuated Capn3 expression in *mdm* mice does not fully explain its phenotype [7, 11]. The same deletion site interrupts binding of CARP/Ankrd1 and ARPP/Ankrd2 proteins to titin, but these proteins, which are associated with hypertrophy signaling are upregulated in *mdm* mice [12], even though muscles do not demonstrate developmental hypertrophy [1]. No sarcomere addition or clear structural changes were observed in *mdm* myofibers [9], but two fiber populations were reported in the *mdm* muscles based on intact striation [8]. This indicates that the disease onset is likely time-dependent or associated with a specific fiber type(s) [8]. Taken together, these reports suggest that signaling and/or functional pathways may also be compromised in *mdm* mice*,* contributing to the extreme phenotypic changes that develop during disease progression [1].

We selected EDL, psoas and soleus muscles for this study since they are the most commonly used muscles in physiological investigations studying fast-twitch (EDL and psoas) and slow-twitch (soleus) muscles [13–15]. They have also been extensively utilized to understand titin’s function [1, 6, 8, 16–19], thus provided a large database of physiological data to utilize in interpreting expression profiles gathered in this study. The three muscles are located in different regions of the mouse body and perform different functions. EDL muscles are predominantly composed of fast-twitch fibers [20] with higher mechanical strength and faster contractile rates, which trigger strong, and more abrupt movements. They are found in the anterior compartment of the leg and associated with foot and toe extension. Psoas is also a fast-twitch muscle that predominantly contains type 2 fast fibers [21]. It is located on either side of the vertebral column connected to the brim of the lesser pelvis and act as flexors and important for maintaining fixed posture in the lumbar spine while supporting the diaphragm movement [22]. The soleus is part of the calf muscles (i.e., *triceps surae*), which acts as the plantar flexors, stabilizing standing posture. Soleus muscles are predominantly composed of slow-twitch fibers that have longer endurance and oxidative capacity.

These three muscles express different titin isoforms with slightly different domain compositions [17, 23], and each muscle exhibits a different degree of response to *mdm* deletion [1, 23]. We compared the expression profiles of wild type EDL, psoas and soleus in a previous study and built a detailed comparison using RNA-sequencing [24]. Here, we are extending that study to investigate molecular level changes behind the complex *mdm* disease phenotype. As the primary cause of the *mdm* phenotype is linked to a deletion in titin, further understanding on this complex disease model will provide a unique opportunity to study titin function beyond sarcomeregenesis and force generation [25].

## Results

To investigate the transcriptomic changes triggered by *mdm* deletion*,* and to understand common and tissue-specific responses to the disease*,* RNA-Seq data collected from EDL, psoas, and soleus were analyzed. Gene expression profiles of *mdm* were compared to wild-type counterparts in each skeletal muscle using DESeq2, and the differentially expressed genes were identified (*p*-adj < 0.01 and absolute fold change > 2).

### *Mdm* muscles carry a distinct expression signature different from wild type muscle

**Fig. 1:**
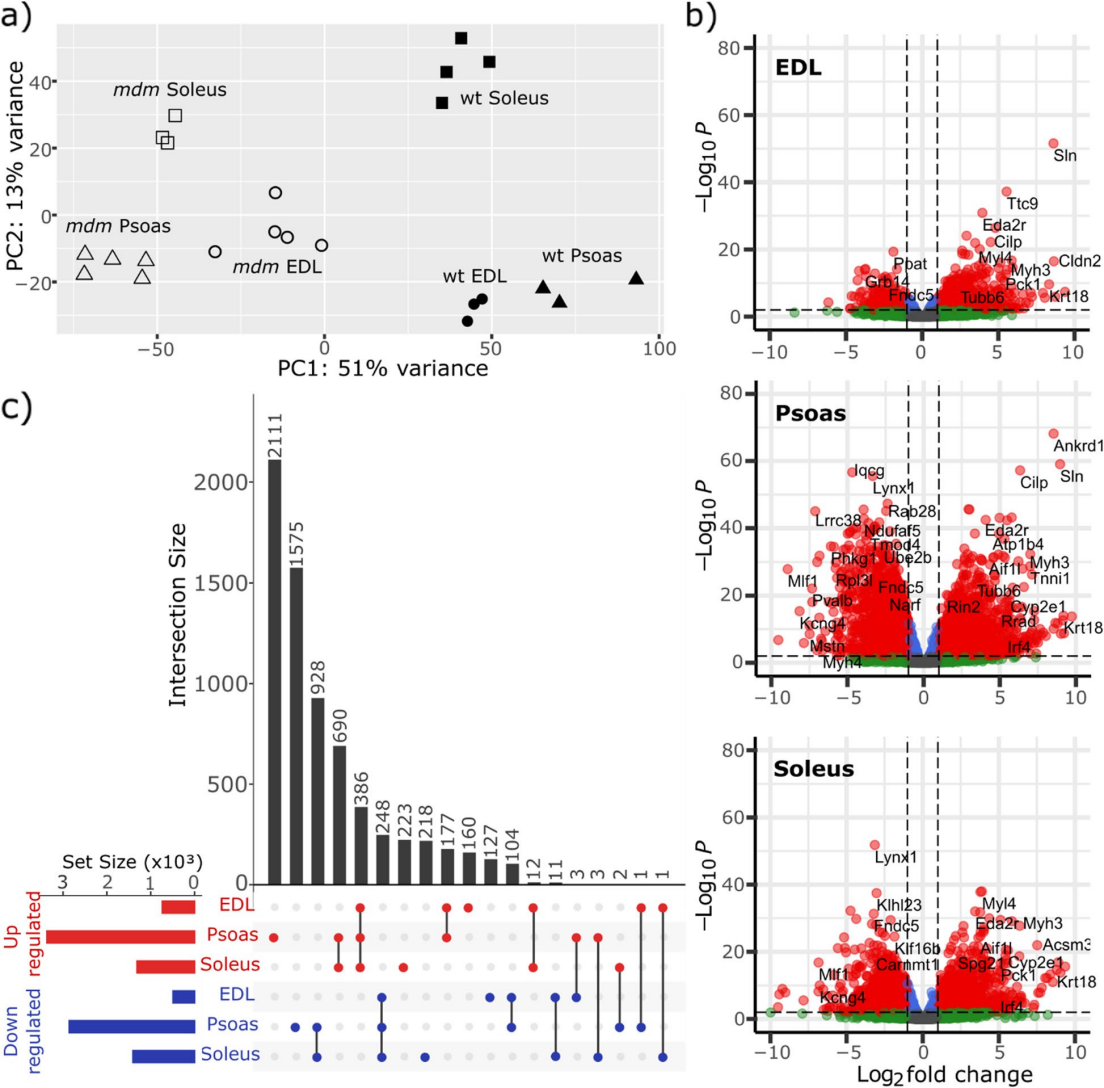
*Mdm* and wild-type muscles clusters separate in the transcriptomics space. **a** Distribution of muscles across Principal Components 1 and 2. The *mdm* and wild-type samples form two distinct clusters along PC1, showing unique transcriptomic characteristics associated with each genotype. **b** Volcano plots show expression profiles of EDL, psoas, and soleus muscles. Red indicates the genes with *p*-adj < 0.01 and absolute fold change >  = 2 in *mdm* muscles compared to wild-type. Gene expression profiles of the three muscles are unique and the magnitude of gene expression changes are not identical. **c** UpSet plot showing muscle specific response to *mdm* mutation in EDL, psoas, and soleus. The number of unique and overlapping genes among the up- and downregulated genes in *mdm* EDL, psoas, and soleus are shown here. The two largest gene subsets are unique to psoas. The next largest gene subsets show the overlap between the up- and downregulated genes between psoas and soleus. Only 386 upregulated genes and 248 downregulated genes were common to all three muscles. Compared to other genes subsets, only very few genes are mutually affected in the two fast muscles (EDL and psoas)

The overall expression pattern shows a well-differentiated *mdm* genotype compared to wild-type muscles. After filtering for marginally expressed genes, principal component analysis (Fig. 1a) showed clear clustering along the first and second principal components (PC), which coincided with both muscle categories and the two genotypes. PC1 explained 51% of the total variance in the expression dataset, which created two primary clusters separating *mdm* samples from wild type. Major grouping along the second principal component (PC2) separated the slow muscle soleus from the fast muscles (EDL and psoas) in both genotypes. This separation was much clearer in wild-type muscles along PC2 but was also present in *mdm* muscles. The results suggest that the muscles specific characteristics are still maintained to some extent in *mdm* muscles despite the phenotypic changes triggered by the *mdm* deletion.

Genes were ranked based on their respective loading values to identify those with the highest contribution to sample grouping along PC1 and PC2. The top 20 genes with the highest contribution to each PC are shown in Table S1. Genes with the strongest association to PC1 affected multiple functionally related gene groups associated with cell adhesion, signaling, muscle contraction, immune/inflammatory response, and metabolism, which implies widespread effects in *mdm* muscles. In contrast, genes most closely aligned with PC2 were those that code for proteins associated with sarcomere structure and function. Most of the genes in this subset encode proteins associated with slow-twitch isoforms, where only 5/20 genes were differentially expressed in *mdm* EDL and *mdm* soleus, while 16/20 genes were affected in *mdm* psoas, indicating a stronger response in psoas compared to EDL and soleus.

Differences among the gene expression profiles of *mdm* EDL, psoas, and soleus were further identified by volcano plots (Fig. 1b) and UpSet plot of unique and overlapping genes among the up- and down-regulated genes identified from each *mdm* and wild-type comparison (Fig. 1C). Of the three muscles investigated, psoas showed the strongest response to the *mdm* deletion. Under the used selection criteria (*p*-adj < 0.01, absolute fold change > 2), 737 upregulated genes and 493 downregulated genes were detected in *mdm* EDL compared to wild-type EDL. Similarly, in *mdm* psoas, there were 3370 upregulated genes and 2858 downregulated genes detected, and in *mdm* soleus, 1313 upregulated genes and 1409 downregulated genes were identified. The UpSet plot shows that overlap among the gene subsets was limited compared to the number of unique genes that were differentially expressed in *mdm* and wild-type psoas. Furthermore, the number of overlapping genes between up- and down-regulated gene sets of *mdm* psoas and soleus was greater than that of EDL and psoas, even though the latter comparison is between fast-fiber-rich muscles. These results support the hypothesis that the *mdm* deletion triggers a specific response in each muscle, rather than a series of uniform responses.

### Muscle specific expression changes in Myosin heavy chains, ARPP, DARP, Titin, and Myopalladin coding genes

**Fig. 2:**
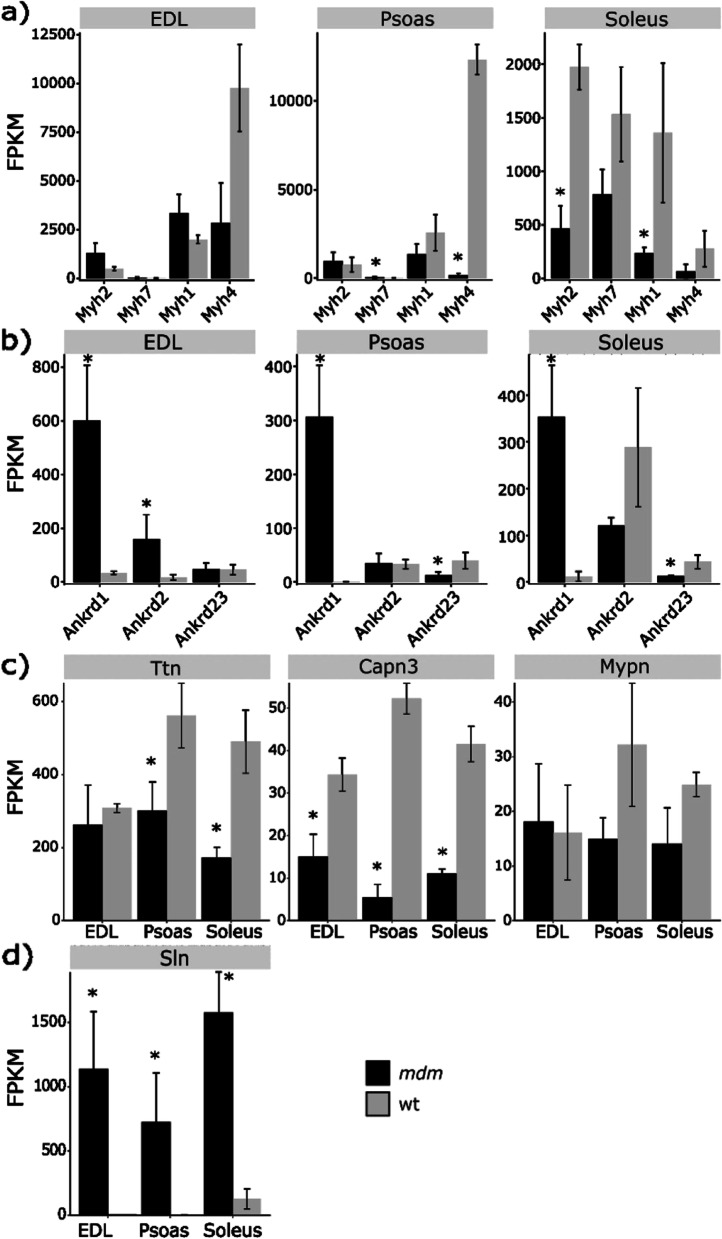
Expression variation of myosin heavy chain (Myh), muscle ankyrin repeat proteins (MARP-CARP(Ankrd1)/ARPP(Ankrd2)/DARP(Ankrd23)), titin (Ttn), Caplain 3 (Capn3), myopalladin (Mypn), and Sarcolipin (Sln) genes in *mdm* vs wild-type muscles. **a** oxidative myosin heavy chains were preferentially expressed over glycolytic myosins in *mdm* EDL, psoas, and soleus but no significant expression difference was found in EDL at the 99% significant level. **b** CARP/Ankrd1 was > 18.7 fold upregulated in all three muscles. A significant expression difference of ARPP/Ankrd2 was only observed in *mdm* EDL (*p*-adj < 0.01). DARP/Ankrd23 was downregulated in *mdm* psoas and soleus. **c** Titin was downregulated in both *mdm* psoas and soleus, but no significant expression difference was observed in *mdm* EDL at the 99% significant level. A 2.8-fold downregulation in Capn3 was observed in *mdm* EDL, psoas and soleus, but Mypn expression was consistent between genotypes. (*p-adj < 0.01). **d** Sarcolipin shows the largest expression change in fast muscles (log2 fold changes – EDL (8.6), psoas (8.9), soleus(3.9))

The myosin heavy chain expression pattern in *mdm* muscles was compared to wild-type muscles for indications of fiber type switching, as previously reported in *mdm* diaphragm muscles [16]. Our results demonstrate that the *mdm* mutation impacts expression of MYH isoforms in a highly muscle-dependent manner (Fig. 2a). Significant expression differences in major MYH isoforms were not observed in EDL (significant level 0.01), even though the Myh4 (which encodes the fast glycolytic MYH-2B isoform) showed a trend towards downregulation. However, *mdm* psoas showed significant upregulation of Myh7 (a slow myosin isoform) and downregulation of Myh4 genes compared to wild-type expression levels. Likewise, in *mdm* soleus, Myh1 and Myh2 showed significant downregulation compared to wild-type soleus. Slow twitch fibers rely on more oxidative metabolic pathways while fast-twitch muscles use more glycolytic metabolism. The collective effect of the observed changes suggests a tendency to express myosin heavy chains that have a more oxidative phenotype in *mdm* muscles. Hessel et al. [23] suggested that major changes in Myh expression profiles in *mdm* muscles were unlikely due to absence of any significant alterations in twitch:tetanus ratio or maximum shortening velocity between the genotypes, consistent with our observations, where the expression changes in MYH isoforms were not universally consistent across muscles.

Because the *mdm* deletion occurs in the titin (Ttn) binding region for MARP (Ankrd1/CARP, Ankrd2/ARPP, Ankrd23/DARP), myopalladin (Mypn), and Calpain 3 (Capn3), we hypothesized that altering the binding site for these proteins might impact the signaling related to expression of these genes. While the downregulation of Capn3 and the upregulation of CARP/Anked1 were consistent across the three *mdm* muscles, *Ttn*, ARPP/Ankrd2, and DARP/Ankrd23 expression varied among muscles (Fig. 2b, c). *Ttn* and DARP/Ankrd23 were downregulated in *mdm* psoas and soleus, while EDL did not show a significant expression difference. Lower levels of titin mRNA in *mdm* psoas and soleus is likely linked to LINE insertion triggered mRNA destabilization, rather than a direct expression downregulation in the gene [26, 27]. The titin gene also undergoes a series of alternative splicing events due to *mdm* deletion, which are described in a complementary paper [28]. ARPP/Ankrd23 was only upregulated in the *mdm* EDL. Witt et al. [123] have previously reported upregulation in CARP and ARPP transcript levels, and no significant expression differences in Capn3 transcripts in *mdm* muscles. In contrast, lower Capn3 protein levels have been reported for *mdm* muscles in other studies [5, 29], which is thought to result from autolysis of free Capn3 [1, 5]. We suggest that lower Capn3 expression levels likely occur as a response to its autolysis and repression of titin mRNA translation over time since the *mdm* mice used in the current study were comparatively older than the 24-day old mice used by Witt et al. [12]. The younger age of the mice in the Witt study meant that they had not fully developed disease symptoms or the characteristic drop in the active force generation. Myopalladin expression levels showed no significant changes in *mdm* muscles (significance level 0.01).

One of the most interesting observations was intense elevation in Sarcolipin (Sln) expression in *mdm* muscles. Sarcolipin (Sln), a sarcoendoplasmic reticulum calcium ATPase, showed a large upregulation in *mdm* muscles compared wild types (Fig. 2d). This is apparent in the volcano plots, where Sln has the highest positive shift in the two fast muscles, EDL and psoas. This expression change was > 8.62 -log2fold in fast *mdm* muscles compared to that of wild type muscles and it was 3.9 -log2fold in *mdm* soleus compared to wild type soleus.

It is also worth noting that discrepancies between this study and Witt et al. [12] could be arising due to differences in experimental design. They used a heterogeneous mixture of RNA, which was extracted from a pool of quadriceps and triceps surae skeletal muscles. In this study, we analyzed EDL, psoas, and soleus muscles independently, so that we can refine our study into individual muscle level. All the muscle dissections were extracted from older mice exhibiting well-developed *mdm* symptoms.

### Mitochondrial-associated genes are downregulated in *mdm* muscles

**Fig. 3:**
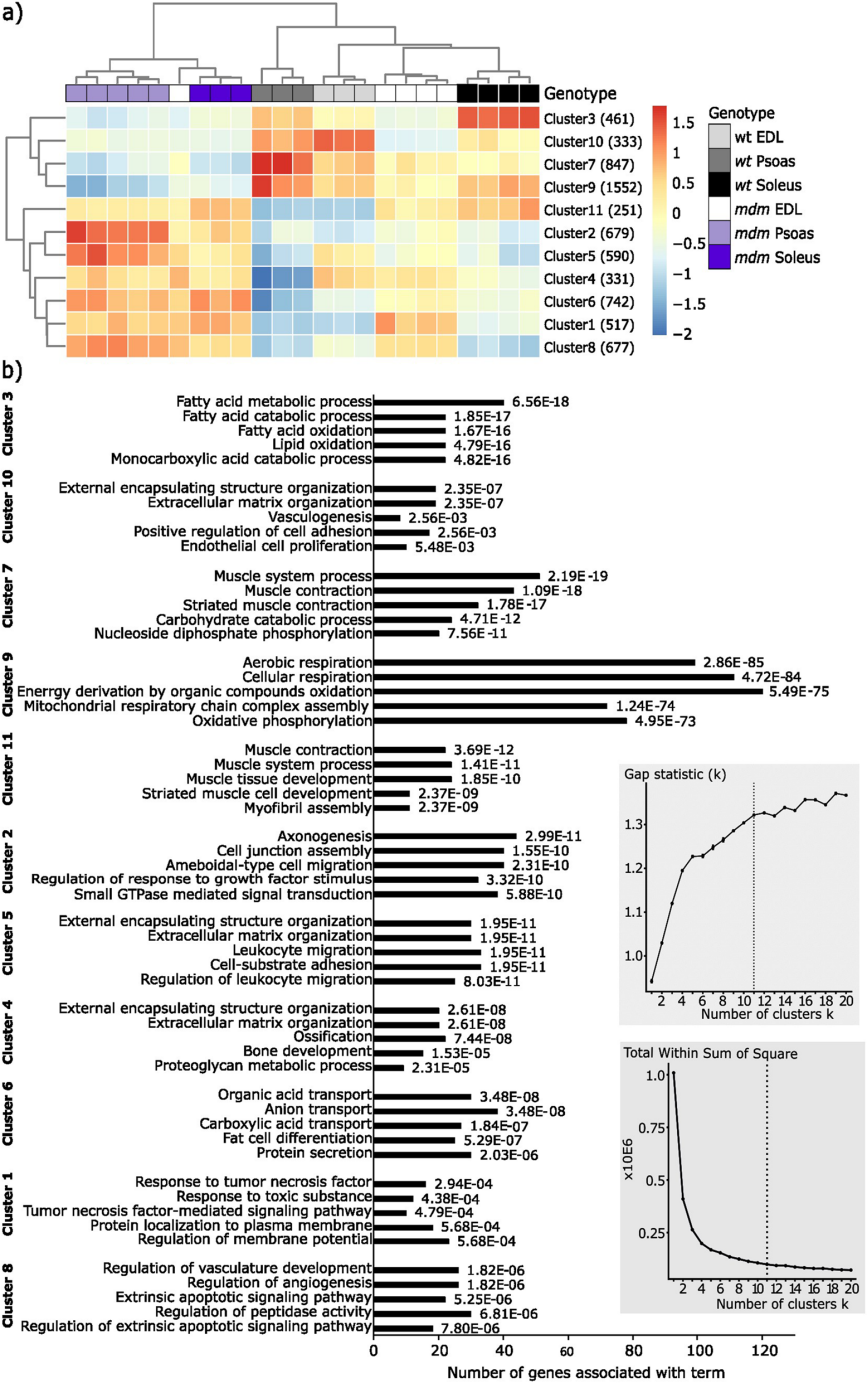
**a** K-means clustering of 6980 differentially expressed genes (*p*-adj < 0.01; absolute log2 fold change >  = 1) identified from mdm vs wild type comparisons in EDL, psoas and soleus. **b** Top 5 GO terms associated with each cluster, when sorted by adjusted *P*-value (shown at right to each bar). Each cell in the clustering heatmap is colored based on the respective Z-value associated with cluster mean. Red = higher expression levels compared to mean expression of gene; Blue = lower expression levels compared to the mean expression of the gene. Optimal number of k-means clusters was determined as 11 based on the maximum gap statistics and minimum within sum of square values calculated for k = 1 to k = 20 (insets)

To understand the cellular functions associated with the differentially expressed genes (DEG) identified from each *mdm* and wildtype comparison, we subset out the DEGs from the expression gene set and k-means clustering was carried out (Fig. 3a). Subsequent gene ontology analysis for each cluster is summarized and shown in Fig. 3b (Full analysis is found in SI table S4). Simultaneously, the DEG sets from each muscle were separated out into up- and down-regulated genes, and mapped to ontology terms. The GO terms summaries for each subset are shown in Figure S1 (*mdm* EDL), Figure S2 (*mdm* psoas), and Figure S3 (*mdm* soleus). The obtained results demonstrated that signal transduction, structural components of myocytes, cellular development, and morphogenesis were among the most upregulated terms associated with all three muscles (Figure S3a, S4a, S5a). Associated signal transduction pathways (Figures S4 and S5) show that the p53 signaling pathway was associated with upregulated genes identified from all three muscles. This pathway is activated as a stress response to cellular events like DNA damage, impairments in the thermal regulation or hypoxia [30] and the latter event is linked to cell cycle arrest, cellular senescence, or apoptosis.

A clear association of downregulated genes in *mdm* with mitochondrial genes was observed in the GO analysis (Figs. 3, S1b, S2b, S3b). Miyano et al. [6] predicted weaker oxidative energy metabolism in *mdm* mice and pathways analysis of the downregulated genes in our study indicated the potential for similar changes in cellular energy status in all three muscles (Figure S5). A majority of the downregulated genes in *mdm* psoas were associated with the TCA cycle and respiratory electron transportation, glycolysis, gluconeogenesis, pyruvate metabolism, ATP synthesis by chemiosmotic coupling, heat production by uncoupling proteins, oxidative phosphorylation, electron transport chain, and thermogenesis (S2b, S3b). In contrast, the downregulated genes in *mdm* EDL were related to glycogen metabolism, collagen biosynthesis, and nucleotide metabolism (Figure S1b), and no direct link to oxidative energy metabolism was observed.

Genes associated with ECM organization and collagen biosynthesis were enriched in the downregulated genes of *mdm* EDL (Fig. 3, Figure S1b), while ECM related genes were upregulated in *mdm* psoas and soleus (Fig. 3, Figures S2b, S3b). These observations are especially interesting as the ECM-associated genes that show differential expression in the two fast muscles (EDL and psoas) have opposing gene expression patterns in response to the *mdm* deletion*.* K-means clustering shown in Fig. 3 highlights the complex expression pattern in ECM associated genes (Clusters 4, 5 and 10) among three *mdm* muscles, and the repression in cellular energy and respiration associated gene expression levels in *mdm* muscles.

Given the evidence of titin’s link to mitochondrial bioenergetics [31], and predictions made by Miyano et al. [6] on impaired oxidative phosphorylation in *mdm* muscles based on reduced thermogenic capacity, along with significant downregulated in oxidative respiration and cellular energy homeostasis associated genes in the current study, we expanded our investigation of mitochondrial association in *mdm* muscles.

### Impairments in mitochondrial respiratory complex1 and mitochondrial proteins synthesis play a central role in *mdm*

**Fig. 4:**
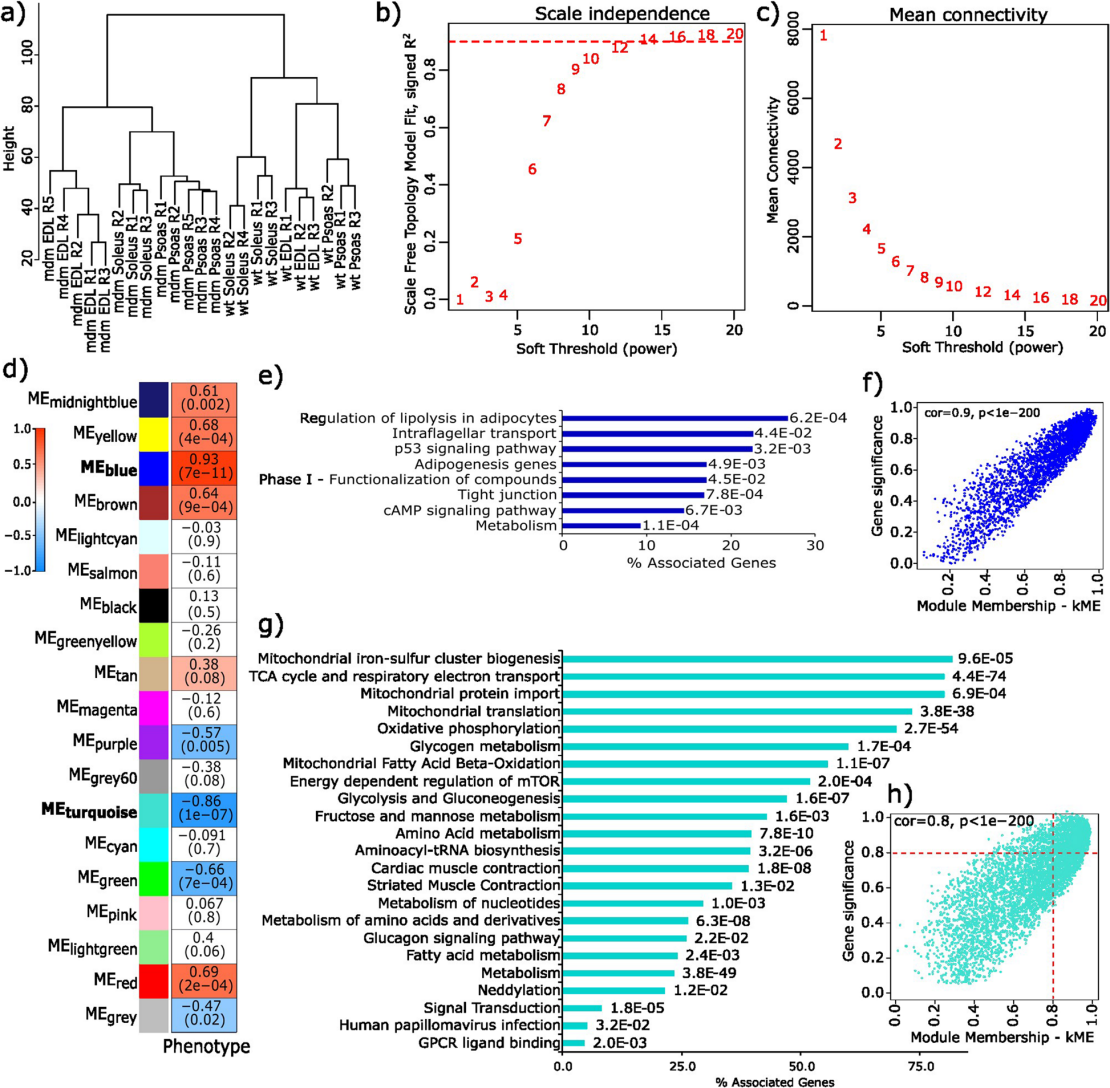
Genes associated with cellular energy metabolism are correlated with the mdm genotype and are highly interconnected. **a**) Sample clustering shows no outlies in the dataset. Two major clusters separated mdm from WT samples, while replicates of each muscle group cluster with each other. **b**-**c**) Power screening for network construction. **b**) Scale free topology (network independence) fluctuation, and **c**) mean connectivity variation based on different power values. **d**) Heatmap of module eigengenes (ME) correlation to the genotype (mdm/wild type). In each cell, the p-values associated with the calculated correlation are enclosed in parentheses. Overview of pathway analysis associated with the differentially expressed genes in **e**) blue, and **g**) turquoise modules. Bars show the percentage of genes associated with each enriched pathway in the module as a fraction of total annotated genes. Adjusted p-value of selected terms are shown at the right side of the bars. Distribution of individual gene significance to genotype, and module membership of genes in **f**) blue, and **h**) turquoise module

To identify primary drivers of strong directional expression response in energy metabolism-associated functions, a weighted gene co-expression network analysis (WGCNA, [32]) was carried out (Fig. 4). Based on the similarity of the expression profiles, 19 gene modules were identified overall with four modules (turquoise, blue, yellow, and red) having module significance > 0.5 and these modules were identified as highly correlated to the *mdm* genotype. Out of four selected modules, eigengenes of the blue and turquoise modules showed a higher absolute correlation to the disease genotype (blue = 0.93, turquoise = -0.86, yellow = 0.68, and red = 0.69), and therefore these modules were selected for additional analysis. Pathway analysis was carried out only for the differentially expressed genes in the blue and turquoise modules (*p*-value = 0.01) as we were particularly interested in identifying altered expression patterns having strong correlation to the *mdm* mutation*.*

**Fig. 5:**
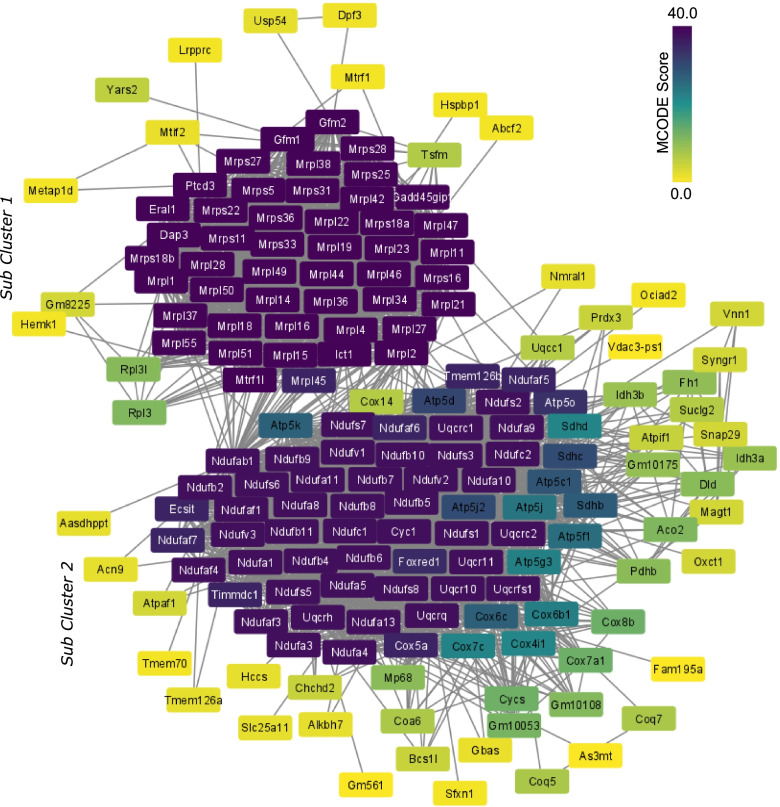
Mitochondrial respiratory complex I and ribosomal protein synthesis associated genes are the putative drivers of the poor energy status of *mdm* muscles. Hub genes identified from the protein–protein interaction (PPI) network were generated for the differentially expressed genes in the turquoise module with gene significance > 0.8, and the module membership > 0.8. The PPI network was created using STRING database. Dark blue denotes highest connectivity, and yellow stands for the least connectivity in the selected hub genes. Colors are based on the connectivity scores calculated by MCODE application in Cytoscape

Gene expression profiles from turquoise module showed a strong negative correlation to *mdm,* and the metabolism and energy homeostasis associated pathways were the primary source of this correlation. To identify the core genes responsible for this outcome in the turquoise module, hub genes with the highest connectivity were identified based on the individual gene significance to genotype (GS > 0.8) and the module membership (kME > 0.8). A protein–protein interaction network was created for the differentially expressed hub genes to identify putative genes having a strong effect on impaired energy metabolism in *mdm* mice. The resulting network was highly populated with 1510 nodes and 5516 edges, so this data set was further reduced to identify the most connected nodes in the network using MCODE. The genes in this subnetwork were chosen as the control centers with a strong bearing on impaired energy metabolism in *mdm* mice (Fig. 5).

**Fig. 6:**
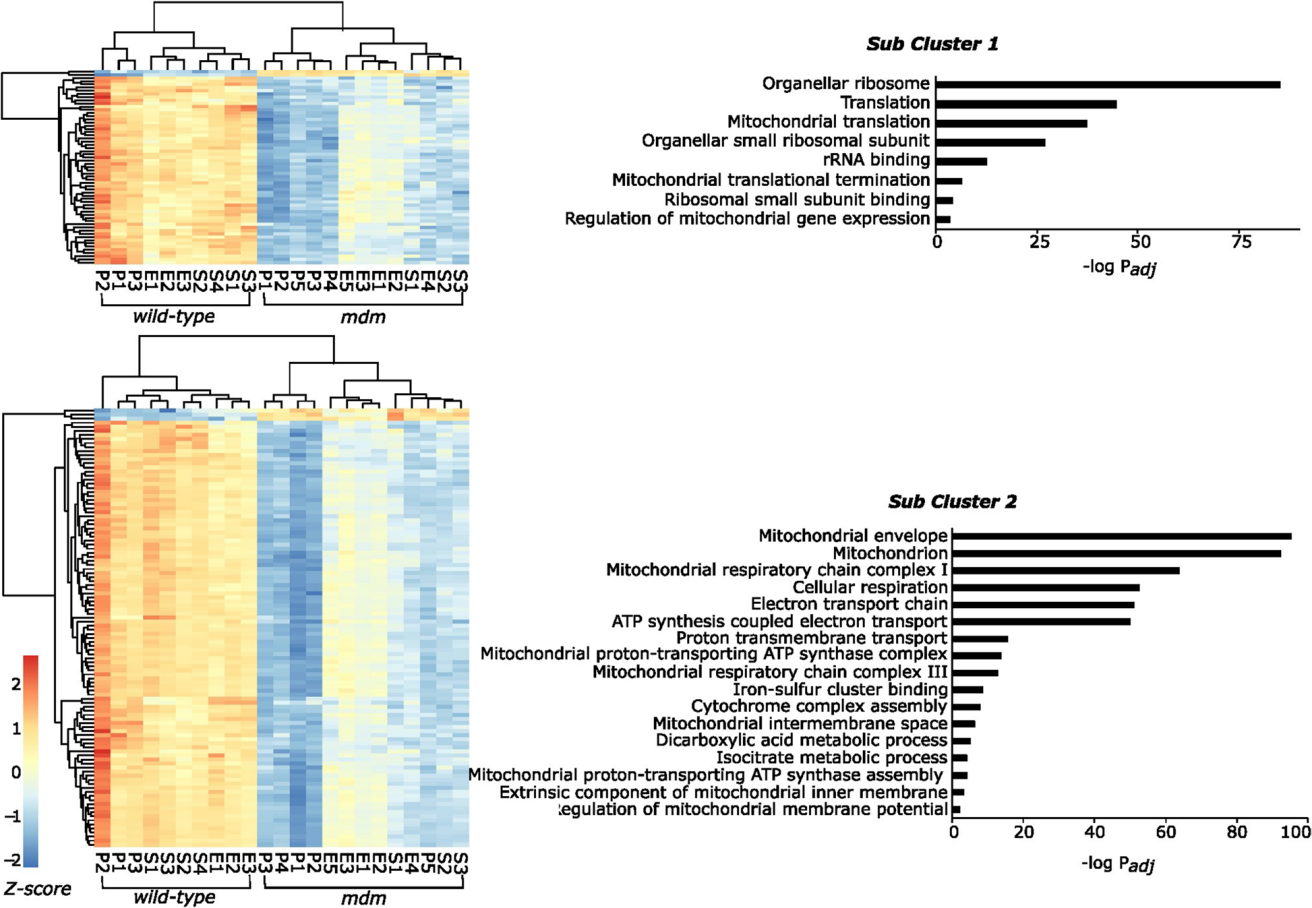
Expression heatmaps and gene ontologies associated with the most connected hub genes in the turquoise module based on their protein-protein interactome. The genes and their connectivity in the protein-protein interaction network is shown in Fig. 5. Genes in the Sub Cluster 1 were associated with mitochondrial protein translation, and the genes in the Sub Cluster 2 were enriched in mitochondrial energy metabolism associated GO terms. Gene ontology analysis was carried out using the ClueGo application in Cytoscape (*p*-adj <0.05). All selected genes showed a clear downregulation in mdm muscles, where psoas showed the most significant response. The column names at the bottom of the heat maps identifies the sample replicates; E-EDL, P-psoas, and S-soleus, followed by the replicate number

The genes in the selected subnetwork can be grouped into two classes based on function: 1) mitochondrial ribosome and translation; or 2) mitochondrial respiration and respiratory complex assembly. Most of the genes in the first group were mitochondrial ribosomal protein-coding genes (MRP), and the second group was populated with the NADH: ubiquinone oxidoreductase subunit (mitochondrial respiratory chain complex I) associated protein-coding genes (NDU), Genes in sub-cluster 2 were enriched in mitochondrial oxidative phosphorylation complex I (NADH: ubiquinone oxidoreductase complex), mitochondrial respiratory chain complex III (Ubiquinol-Cytochrome C Reductase subunit), and mitochondrial respiratory chain complex V (ATP Synthase Subunit). Gene ontologies and expression heatmaps associated with each gene subset are shown in Fig. 6. The above observations align with mitochondrial myopathies primarily caused by defects in the respiratory mechanism due to enzyme deficiencies or structural changes [33].

## Discussion

Muscular dystrophy with myositis (*mdm*) is an important mouse model to study titin function in skeletal muscles as it affects a key active stiffness modulator in activated muscles [1, 25]. Although symptoms overlap with many common muscle diseases, the molecular signatures seen in *mdm* muscles do not align with common phenotypic changes found in muscular dystrophy [1]. The current study attempts to improve our understanding of the *mdm* phenotype as means of improving our understanding of titin’s function and titinopathies.

There was no evidence to demonstrate a severe expression shift in MYH isoforms in *mdm* EDL or soleus, suggesting that the *mdm* mutation does not trigger fiber type switching. This observation indicates that *mdm* mutation induced changes are highly subjective to each skeletal muscle and are consistent with the findings of Hessel et al. [23] and Powers et al. [8]. In contrast, we observed consistent expression shifts in Ankrd1/CARP and Capn3 genes in *mdm* muscle compared to wild type levels, that are similar to the previous reports [12, 34], highlighting the importance of these genes in disease development.

The major observation of the study was that there was a strong response in mitochondrial protein coding genes, especially associated with protein complexes in mitochondrial respiratory chain in *mdm* muscles. *Mdm* mice show poor thermoregulation and frequent muscle tremor at ambient temperatures as well as severe impairments in both shivering and non-shivering thermogenesis [6, 10]. Heat produced from non-shivering thermogenesis, shivering thermogenesis, and futile calcium cycling contribute to the thermoregulation in skeletal muscles of endotherms, for which mitochondrial processes also plays an important role [33, 35]. Our results provide new insights into these previously observed deficiencies and their possible association to *mdm* pathology.

Of all the genes undergoing expression changes in the presence of the *mdm* deletion, the sarcolipin (Sln) gene showed the largest upregulation in fast *mdm* muscles compared to WT. Sln is an important intermediate transporter in futile Ca^2+^ transfer necessary for heat generation in muscle [36] and plays a role in both shivering and non-shivering thermogenesis [37, 38]. Expression of the Sln gene is developmentally regulated in rodents. Higher Sln levels are found in neonatal muscles, but expression decreases with age, particularly in fast muscles [36]. Upregulation of Sln in muscle diseases is a compensatory mechanism for higher energy demands and is linked to mitochondrial biogenesis and oxidative metabolism [22]. Higher level of Sln, similar to those reported here, were reported in a Duchenne muscular dystrophy (DMD) mouse model and in muscle atrophy, especially in glycolytic muscles [39]. Even a minor drop in the body temperature is enough to trigger expression of Sln in skeletal muscles, which acts as a marker for cellular efforts to improve muscle energetics and fatigue [38]. Based on the above evidence, we suggest that upregulation of Sln is an adaptive response to impaired thermoregulation in *mdm* muscles.

We observed a strong negative response in gene expression related to cellular energy metabolism and mitochondrial heat production, especially in psoas and soleus muscles. Differential gene expression analysis and the subsequent WGCNA showed a strong correlation between *mdm* genotype and genes associated with oxidative phosphorylation, glycolysis, and metabolism (Fig. 4). Most connected genes (hub genes) identified from the gene co-expression network analysis and the subsequent PIP network point to mitochondrial complex I, III and V coding genes (Fig. 6), which likely arise and accumulate at later stage in disease pathology. However, evidence supporting Capn3 deficiency being associated with poor mitochondrial health elsewhere [40] is an interesting detail that could imply a direct link between the strong response in mitochondrial associated genes to *mdm* mutation.

The second subset of hub genes identified from the PPI network encodes for proteins that are engaged in mitochondrial protein synthesis, where a majority of the genes were mitochondrial ribosomal protein (MRP) coding genes. Dysregulation of proteins associated with mitochondrial protein synthesis has been identified as a critical factor affecting oxidative phosphorylation [41]. Defects in mitochondrial energy production affect cell growth and development, and MRP deficiencies are linked to mitochondrial diseases [42]. Sarcopenia (muscle loss with age) and sedentary behavior are often accompanied by weak mitochondrial protein synthesis, lower enzyme levels associated with oxidative phosphorylation and electron transport chain, and impaired mitochondrial function [43]. Mitochondrial health is measured through metabolic indicators [43], thus the reduced metabolic rates observed in *mdm* mice [6] may associate with weaker mitochondrial health*.* Upregulation of p53 signaling also suggests the possibility of increased oxidative stress, and thus mitochondrial dysfunction [44], in *mdm* muscle.

Impairments in mitochondrial respiratory chain complexes are commonly observed with the progression of neuromuscular disorders [45]. While the *mdm* phenotype is not directly linked to mitochondrial genes, the downregulation of mitochondrial respiratory complex associated genes may occur as a secondary effect of the *mdm* deletion as discussed above. As we have observed *mdm* muscles having a propensity to develop a slow fiber type environment, and slow fibers have a higher demand for oxidative energy, the impairments in mitochondrial respiratory complex may interfere with complete of fiber type switching. This may explain why different studies have suggested contradicting models for slow myosin expression in *mdm* mice [9, 16, 23]. It is possible that *mdm* muscles contain a heterogenous mixture of fibers at different stages of disease development, that may lead to mixed signals in whole muscle investigations.

This study lays out a strong set of transcriptional changes seen in *mdm* muscles. Even though the primary genomic mutation associated with *mdm* is mapped to the Ttn gene, we were unable to draw evidence supporting titin as the primary culprit in the overall disease pathology. This suggests that the downregulation in titin gene in *mdm* psoas and soleus may be linked to mRNA destabilization due to the L1 retrotransposon-insertion in the primary mutation site. Such mutational events may prompt genomic and mRNA instability, which in turn leads to lower titin mRNA levels in *mdm* muscles, which could be identified as downregulation in gene expression. Insertional mutations by L1 retrotransposons are reported to be linked to muscle dystrophy in humans [46], and one such intronic insertion is found in dystrophin gene and among the mutational events causing DMD in humans [47].

## Conclusions

*Mdm* is primarily associated with a small deletion in the titin gene and thus *mdm* mice are an important model for studying titin’s function in skeletal muscles. In this comparative transcriptomic study, we were able to identify a series of complex transcriptomic changes that occur in *mdm* muscles. Response to the *mdm* deletion is highly variable among skeletal muscles, even within the same muscle-type category, unlike many common muscle diseases. Hence a uniform response pattern cannot be identified nor can be generalized based on the presence of the *mdm* deletion. We provide evidence showing severe impairments in the mitochondrial oxidative respiration and energy homeostasis in *mdm* muscles. Mitochondrial oxidative phosphorylation complexes I, III, and V associated genes and mitochondrial ribosomal protein-coding genes showed the strongest expression rearrangement due to *mdm* mutation at the climax of disease accretion. Strong upregulation seen in the sarcolipin gene further supports these claims. We also propose that the LINE-1 retrotransposon insertion in titin gene destabilizes its mRNA, which in turn disconnects titin from strong expression changes observed in cellular respiration and energy homeostasis in *mdm* muscles.

## Materials and methods

### Sample preparation

Muscle tissue was obtained from heterozygous B6C3Fe a/a-Ttn*mdm*/J mice originally obtained from the Jackson Laboratory (Bar Harbor, ME, USA) to establish a mouse colony in the animal care facility of Northern Arizona University, Flagstaff, AZ, USA. Mice were maintained at 12 h:12 h light: dark cycle in a temperature-controlled facility and fed ad libitum. The use of animals and the experimental protocol were approved by the Institutional Animal Care and Use Committee at NAU (Reference number:18–002). The number of mice used for the study was based on the availability of the required muscles as the samples were collected from mice euthanized for other experimental studies.

Whole muscles were surgically removed from mice between 29–54 days old. Three biological replicates for wild-type EDL, wild-type psoas, and *mdm* soleus, four biological replicates for wild-type soleus, and five replicates for *mdm* EDL and *mdm* psoas muscles were isolated from a total of 14 conscious mice sacrificed under 0.5 ml of isoflurane gas in a euthanization chamber. After extraction, muscles were stored in RNAlater™ stabilization solution (ThermoFisher Scientific) at -80 °C until RNA extraction was conducted.

### RNA extraction and next-generation sequencing

Total RNA from the collected muscles was extracted according to the recommended protocol in the Qiagen Fibrous Tissue Total RNA extraction mini kit. RNA concentration was measured using a Qubit RNA Broad-Range assay, and RNA integrity number (RIN) was assessed with Agilent 2100 Bioanalyzer RNA 6000 Nano assay. Samples with a concentration > 20 ng/ul, RIN > 7 were chosen for the subsequent cDNA library preparation. cDNA libraries from the selected samples were prepared with Illumina TruSeq Stranded Total RNA Library Prep Kit. Quality of the cDNA libraries was assessed with KAPA Library Quantification qPCR Kit for Illumina sequencing platforms, and library fragment sizes were tested using an Agilent 2100 Bioanalyzer High Sensitivity dsDNA quantification assay. The mean library sizes of the samples were between 256-323 bp (Tables S2 and S3). Prepared cDNA libraries were sequenced in an Illumina NextSeq 500 high throughput sequencer following sequencing guidelines recommended by Illumina. Short 75 bp paired-end reads were generated from each library, and the samples were sequenced using a total of five sequencing runs. The sequenced library coverage varies between 9.5—100 million reads with a median of 23 million reads (Tables S2 and S3).

### Raw data processing

Post sequencing read quality was checked using the FastQC quality control tool (http://www.bioinformatics.babraham.ac.uk/projects/fastqc/) for high throughput sequence data. If the per-base quality score was below 20 at any position along the 75 bp length stretch, those samples were processed using sliding window quality filtering (window size = 4 bp) in Trimmomatic v0.32 [48]. After filtering, only the paired-end reads collected from the read-trimming were used for downstream data analysis (Table S3). Original samples that showed a satisfactory per base quality score (> 20) were used without filtering. Sequencing adapters were trimmed while converting initial BCL data to fastq files from the sequencing center prior to receiving the data files and no adaptor contamination was detected in FASTQC analysis.

### Data alignment

Prepared fastq files were aligned to the *Mus musculus* GRCm38.p4 genome annotation using the Tophat alignment tool. In order to calculate the insert sizes between paired-end reads, a subset of 250,000 reads from each sample was aligned using the BWA-aln [49] short read alignment tool available on the Galaxy web platform [50]. The built-in reference mouse genome (mm10) was used to carry out the alignment under default settings. Alignment statistics for the pre-alignments were generated using CollectInsertSizeMetrics Picard tool (http://broadinstitute.github.io/picard/), and average insert sizes and standard deviations were fed into subsequent complete read alignments generated with Tophat v2.1.1 [51] (See alignment details in Tables S2 and S3).

### Differential gene expression analysis

Statistical data analysis was carried out with R_v3.6.1. Gene-wise read counts were generated for the alignments using GenomicFeatures v1.34.3 [52] and GenomicAlignments v1.18.1 libraries. Marginally expressed genes were filtered out to increase the precision of the statistical analysis. If a particular gene had a DESeq2 normalized read count of less than 10 in less than three muscles, these genes were identified as marginally expressed, and removed from the dataset. After this filtering step, the dataset contained a total of 17,924 genes (out of 46,078 annotated genes in the reference genome) between *mdm* and wild-type samples. Subsequent analysis used this set of genes, which is identified as the expression dataset hereafter (heatmap of the expression dataset is shown in Figure S6). A distance matrix was generated using R dist function on the variance stabilized transformed (DESeq2::vst) [53, 54], and sequencing batch effect corrected (limma:: removeBatchEffect) expression dataset to check the correlation among replicates (Figure S7). Replicates from each muscle created two unique major clusters that coincide with the wild-types and *mdm* classifications of the muscle samples*.*

Differential gene expression analysis was carried out using R library DESeq2, in which data is fitted using a generalized linear negative binomial distribution model to account for subtle changes in gene expression. Statistical significance of differential gene expression in *mdm* with reference to the wild type was tested in each muscle using the Wald test, as the dataset has been modeled as a binomial distribution (design =  ~ run + group, where group factor levels represent 6 sample categories based on muscles and genotype). Calculated *p-values* were adjusted using the Benjamini–Hochberg multiple testing procedure [55] in the subsequent DESeq2 protocol steps to increase statistical testing accuracy. Genes with *p-value* < 0.01 (a higher significance level (99%) was selected to avoid testing bias due to small sample size), and fold change > 2 were identified as significantly differentially expressed from each *mdm* – wild-type comparison.

### Gene ontology analysis

Gene ontology (GO) analysis for up and down regulated gene subsets was carried out using the ClueGO_v2.5.5 [56] application in Cytoscape [57] (Organism: Mus Musculus [10090], reference sets = GO BP, MF, CC EBI-UniProt-GOA_17.08.2020_00h00, Statistical Test Used = Enrichment/Depletion (Two-sided hypergeometric test), *p*-value cutoff = 0.05, Correction Method = Bonferroni step down, Min GO Level = 3, Max GO Level = 7, Number of Genes = 3, Min Percentage = 10.0, GO Fusion = true, GO Group = true, Kappa Score Threshold = 0.4, Sharing Group Percentage = 50.0). Identified GO terms were functionally grouped in ClueGO using a Kappa score based on shared genes among terms. Each identified group was named based on the GO term with the lowest *p*-value. Since differentially expressed genes from psoas and soleus were enriched in a large set of terms, which was hard to handle, the specificity of analysis was increased by using GO terms supported by experimental and computational evidence codes reviewed by the curator (Evidence codes used: All Experimental (EXP,IDA,IPI,IMP,IGI,IEP), IGC,ISA,ISM,ISO,ISS,RCA).

### Data visualization and graphical output generation

The principal component analysis was carried out using R prcomp function on the variance stabilizing transformed and sequencing batch effect corrected expression dataset. The UpSet plot was created using R library UpSetR_1.4.0 [58]. The bar charts were generated using the R library ggplot2_ 3.3.2 (https://ggplot2.tidyverse.org/). Volcano plots were generated using EnhancedVolcano library (https://github.com/kevinblighe/EnhancedVolcano).

Normalized gene expression levels in Fragments Per Kilobase Million (FPKM) were calculated using DESeq2::fpkm function with default parameters, where gene lengths were determined using the union of all GRanges assigned to a particular gene in the data object (https://www.rdocumentation.org/packages/DESeq2/versions/1.12.3/topics/fpkm).

K-means clustering and subsequent GO analysis for each identified gene cluster were carried out in R_v 4.2.1. Variance stabilizing transformed and sequencing batch effect corrected differentially expressed genes were used as the data input. The optimal number of clusters was determined using elbow and gap statistics methods (NbClust::fviz_nbclust). Heatmap of Z-scores was generated using pheatmap_1.0.12. GO analysis for each gene cluster was conducted using clusterProfiler_4.4.4 package and org.Mm.eg.db_3.15.0 database.

### Weighted gene co-expression network analysis (WGCNA)

A more stringent data selection criterion was used to filter genes from the original dataset to reduce noise and improve module detection [32]. Genes containing more than 10 samples (wild type = 10, *mdm* = 13), with DESeq normalized read count > 10 were chosen for the analysis, which resulted in a data set of 15,649 genes. The filtered data set was prepared for data analysis using variance stabilizing transformation followed by batch effect correction. The prepared dataset was clustered using the R hclust function with an average distance to look for outliers. No outliers were detected as clear clustering was observed among the *mdm* and wild-type samples, and replicates from the same muscles were grouped together within respective genotypes associated clusters. The subsequent analysis was carried out using R library WGCNA as described in the documentation (https://horvath.genetics.ucla.edu/html/CoexpressionNetwork/Rpackages/WGCNA/). An appropriate soft thresholding power (β) was determined based on the scale-free topology criteria recommended by the developers (R^2^ ~ 0.9). A signed network was chosen as the appropriate fit for the analysis as we were interested in identifying biologically meaningful gene modules sensitive to the directionality of gene expression change from the wild-type base levels. For signed network construction, β = 14 was selected using the WGCNA function pickSoftThreshold. The correlation network was created with one step network construction function blockwiseModules in the WGCNA library (power = 14, TOMType = "signed", minModuleSize = 30, networkType = "signed", reassignThreshold = 0, mergeCutHeight = 0.25, numericLabels = TRUE, maxBlockSize = 20,000). Genotype (*mdm* / wild-type) correlation to expression variation of genes in each module was calculated using the WGCNA function cor, and respective significant values were generated using the WGCNA function corPvalueStudent. Modular significance values (kME) were generated using the WGCNA function signedKME for the initial input dataset for the WGCNA, and module eigengenes calculated using the WGCNA function moduleEigengenes.

Average gene significance to genotype was taken as the modular significance (MS), and gene modules with MS > 0.5 were selected. The number of modules was further reduced based on absolute MS, and blue and turquoise modules were selected as they had significantly higher average gene significance. A pathway analysis was carried out on the DEseq2 identified differentially expressed genes for the blue and turquoise modules. To increase the mappability of genes into pathways, KEGG, Wiki, and Reactome pathway annotations were used in ClueGO v2.5.7 in Cytoscape v3.8.1 (*Pvalue* cutoff = 0.05, Correction Method Used = Bonferroni step down).

### PPI network construction and hub gene selection

A protein–protein interaction (PPI) network was created using the STRING online database (https://string-db.org/). As the STRING database input has a maximum node cutoff = 2000, genes with the highest connectivity within the module were selected based on co-expression measures. The module size was reduced using two cutoffs—individual gene significance to genotype (GS > 0.8) and the module membership (kME > 0.8). The differentially expressed genes as identified by DESeq2 in the resulting gene subset were used to create the PPI network with a high confidence cutoff (0.7). The MCODE application plugin in Cytoscape v3.8.1 was used to identify the most connected genes (hub genes) in the PPI (haircut = NO, loop = NO, fluff = 0.5, node score = 0.1, degree cutoff = 2, k-score = 2).

## Supplementary Information

**Additional file 1:** **Figure S1.** Overview of gene ontologies associated with a) up- and b) down-regulated genes in mdm EDL. **Figure S2.** Overview of gene ontologies associated with a) up- and b) down-regulated genes in mdm psoas. **Figure S3.** Overview of the gene ontologies associated with a) up, and b) downregulated genes identified from mdm soleus. **Figure S4.** An Overview of pathways enriched from the upregulated genes identified from mdm and wild-type comparisons in a) EDL, b) psoas and c) soleus. **Figure S5.** An verview of pathways enriched from the downregulated genes identified from mdm and wild-type comparisons in a) EDL, b) psoas and c) soleus. **Figure S6.** Gene expression heat map of the complete data set, after removing marginally expressed genes. **Figure S7.** Distance matrix among transcriptomic profiles show clear separation between mdm and wild-type samples. **Table S1.** Top 20 genes contributing to the variance represented by principal component l (PC1) and principal component 2 (PC2). **Table S2.** RNA-Seq read alignment summary for the wild-type samples used in the study. **Table S3.** Alignment statistics of mdm samples used in the study.

**Additional file 2:** **Table S4.** Full gene ontology of differentially expressed genes (excel file).

## Data Availability

The datasets used and/or analyzed during the current study available from the corresponding author on reasonable request. The raw RNA-Seq data is uploaded to GEO: WT data (Accession Number: GSE158283) and *mdm* data (Accession Number: GSE210263).
