# Supplementary material for: Transcriptomic profiles of muscular dystrophy with myositis (mdm) in extensor digitorum longus, psoas, and soleus muscles from mice
Source: BMC Genomics. 2022 Sep 17;23:657. doi: 10.1186/s12864-022-08873-2 (PMC9482285; doi:10.1186/s12864-022-08873-2)
Supplement: Supplementary file 1 — Additional file 1: Figure S1. Overview of gene ontologies associated with a) up- and b) down-regulated genes in mdm EDL. Figure S2. Overview of gene ontologies associated with a) up- and b) down-regulated genes in mdm psoas. Figure S3. Overview of the gene ontologies associated with a) up, and b) downregulated genes identified from mdm soleus. Figure S4. An Overview of pathways enriched from the upregulated genes identified from mdm and wild-type comparisons in a) EDL, b) psoas and c) soleus. Figure S5. An verview of pathways enriched from the downregulated genes identified from mdm and wild-type comparisons in a) EDL, b) psoas and c) soleus. Figure S6. Gene expression heat map of the complete data set, after removing marginally expressed genes. Figure S7. Distance matrix among transcriptomic profiles show clear separation between mdm and wild-type samples. Table S1. Top 20 genes contributing to the variance represented by principal component l (PC1) and principal component 2 (PC2). Table S2. RNA-Seq read alignment summary for the wild-type samples used in the study. Table S3. Alignment statistics of mdm samples used in the study. [file 12864_2022_8873_MOESM1_ESM.docx]

**Supplementary information**


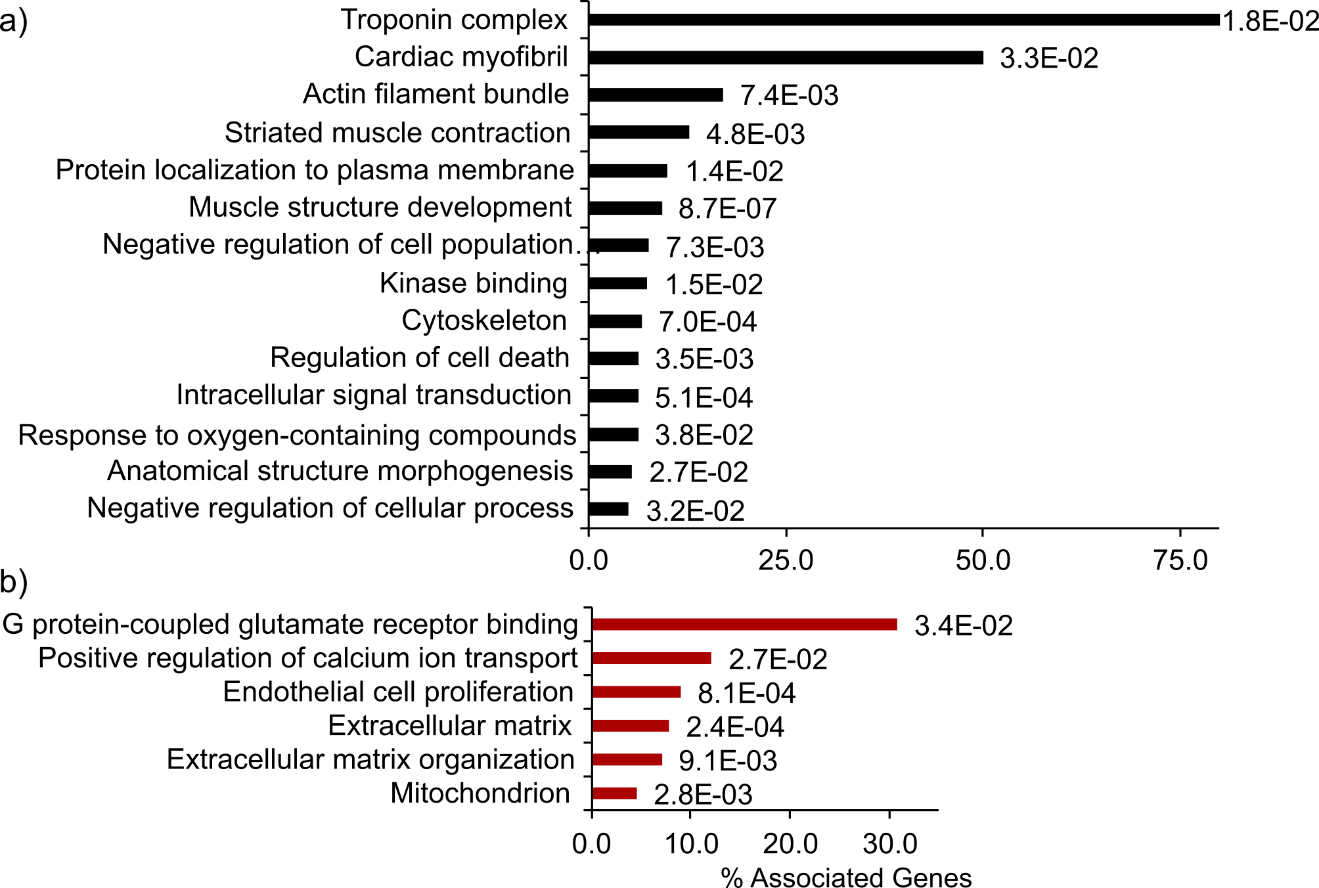


**Figure S1: Overview of gene ontologies associated with a) up- and b) down-regulated genes in *mdm* EDL.** Analysis was carried out in ClueGo application in Cytoscape using all available evidence codes and *padj*<0.05. Bars show percentage of differentially expressed genes associated with each GO term as a fraction of total annotated genes. Terms representing each group of GO terms sharing similar sets of genes are shown. Adjusted P-value of selected terms are shown at the right side of the bars. A total of 356/737 upregulated genes and 88 /493 downregulated genes were mapped to gene ontologies under the selection criteria used.


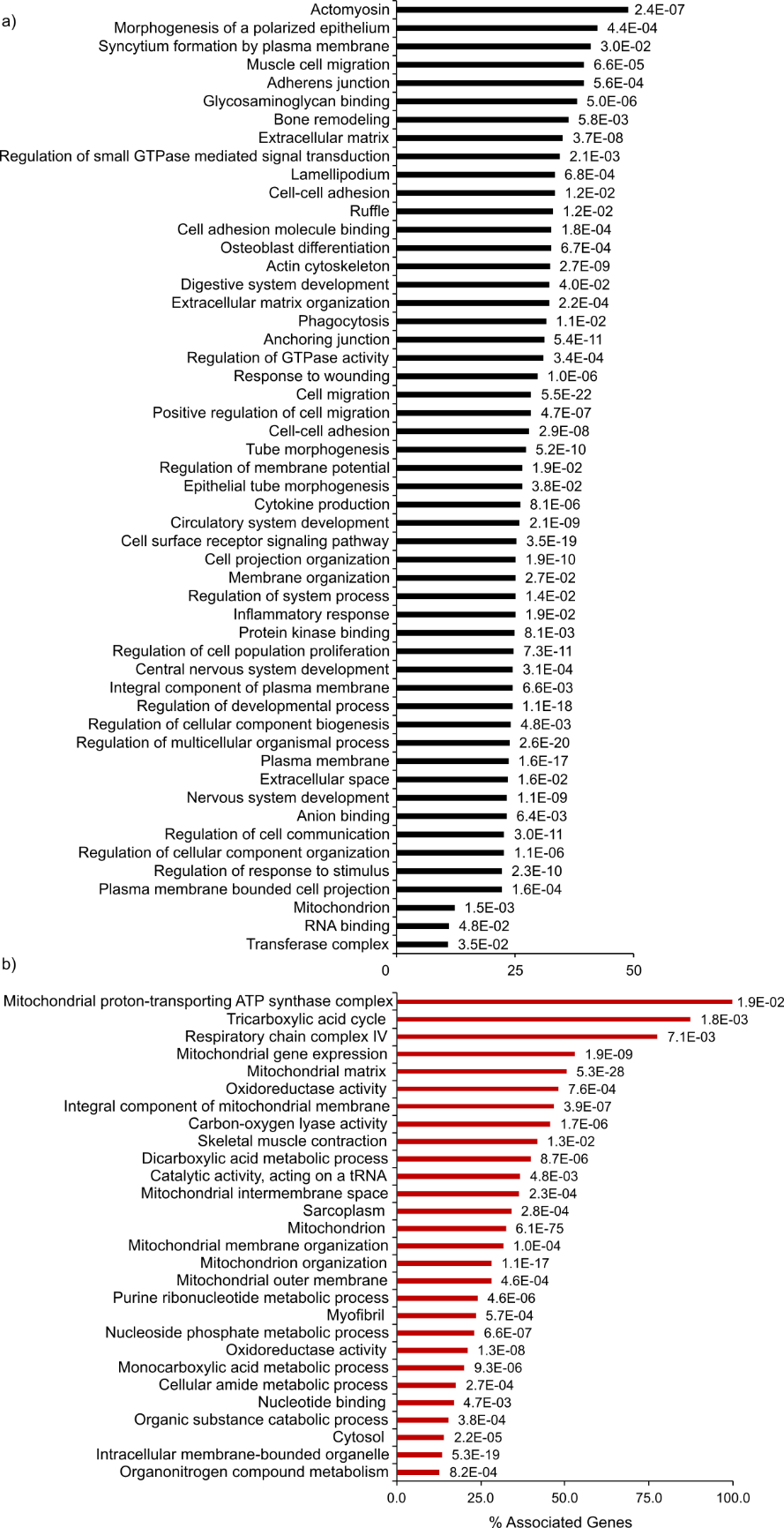


**Figure S2: Overview of gene ontologies associated with a) up- and b) down-regulated genes in *mdm* psoas.** Analysis was carried out in ClueGo application in Cytoscape (evidence codes used: All Experimental (EXP,IDA,IPI,IMP,IGI,IEP), IGC,ISA,ISM,ISO,ISS,RCA; *padj*<0.05). Bars show percentage of differentially expressed genes associated with each GO term as a fraction of total annotated genes. Terms representing each group of GO terms sharing a similar set of genes are shown. Adjusted P-value of selected terms are shown at the right side of the bars. A total of 1961/3370 upregulated genes and 1168/2858 downregulated genes were mapped to gene ontologies under the selection criteria used.


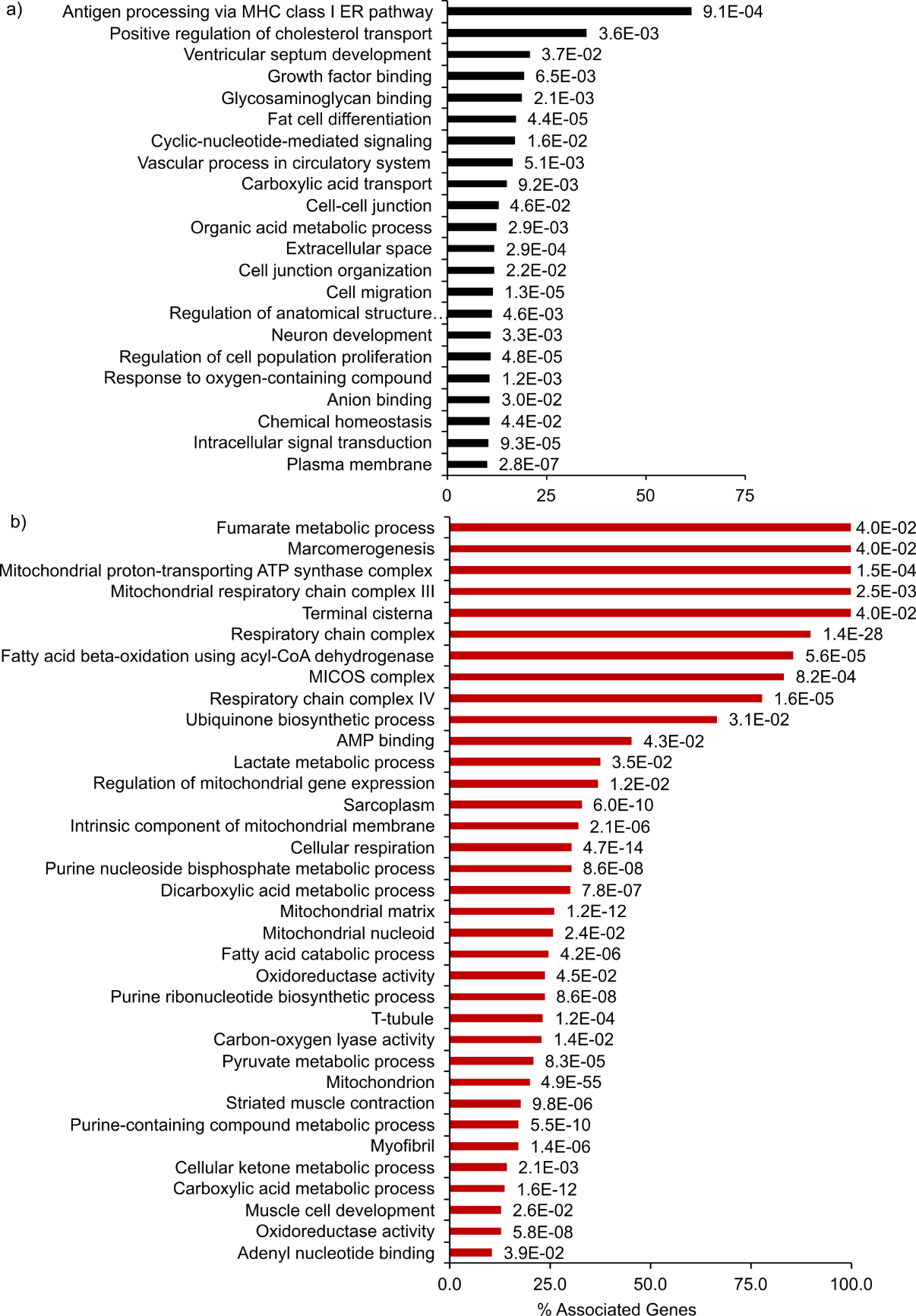


**Figure S3: Overview of the gene ontologies associated with a) up, and b) downregulated genes identified from *mdm* soleus.** Analysis was carried out in ClueGo application in Cytoscape (Evidence codes used: All Experimental (EXP,IDA,IPI,IMP,IGI,IEP), IGC,ISA,ISM,ISO,ISS,RCA; *padj*<0.05). Bars show percentage of differentially expressed genes associated with each GO term as a fraction of total annotated genes. Terms representing each group of GO terms sharing similar set of genes are shown here. Adjusted P-value of selected terms are shown at the right side of the bars. A total of 727/1313 upregulated genes and 410/1409 downregulated genes were mapped to gene ontologies under the selection criteria used.

**
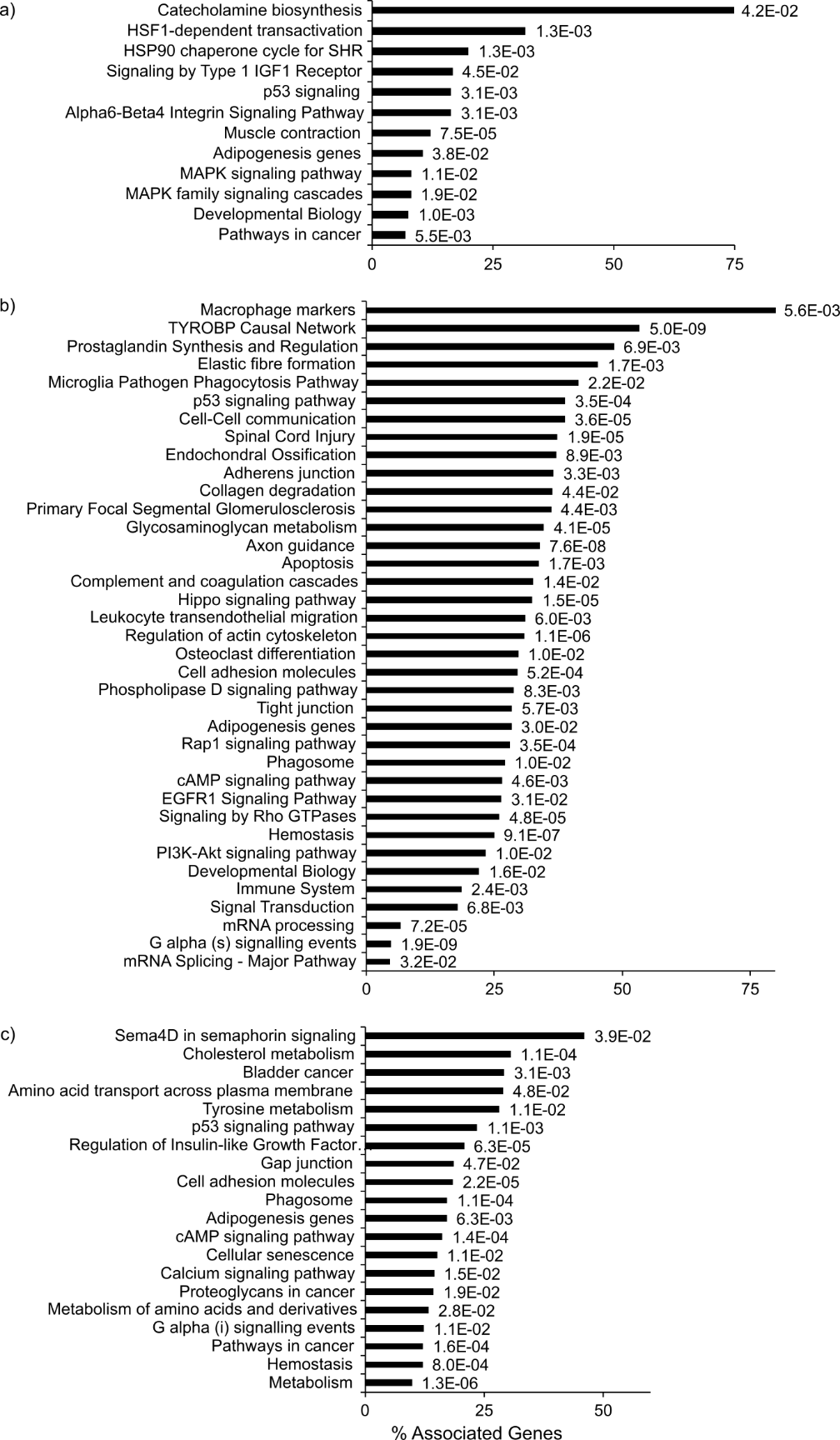
**

**Figure S4: An Overview of pathways enriched from the upregulated genes identified from *mdm* and wild-type comparisons in a) EDL, b) psoas and c) soleus.** Each upregulated gene set was searched against KEGG, Wiki and Reactome pathway annotations using ClueGO tool in Cytoscape application under p-adj cutoff of 0.05. Results were summarized based on the gene overlap among terms, and the representative term for each group was selected based on the p-adj value. The percent associated genes denotes the percentage of differentilly upregulated genes associated with representatative pathways as a fraction of total annotated genes. Adjusted p-values associated with the pathways are shown at the right side of the bars.

**
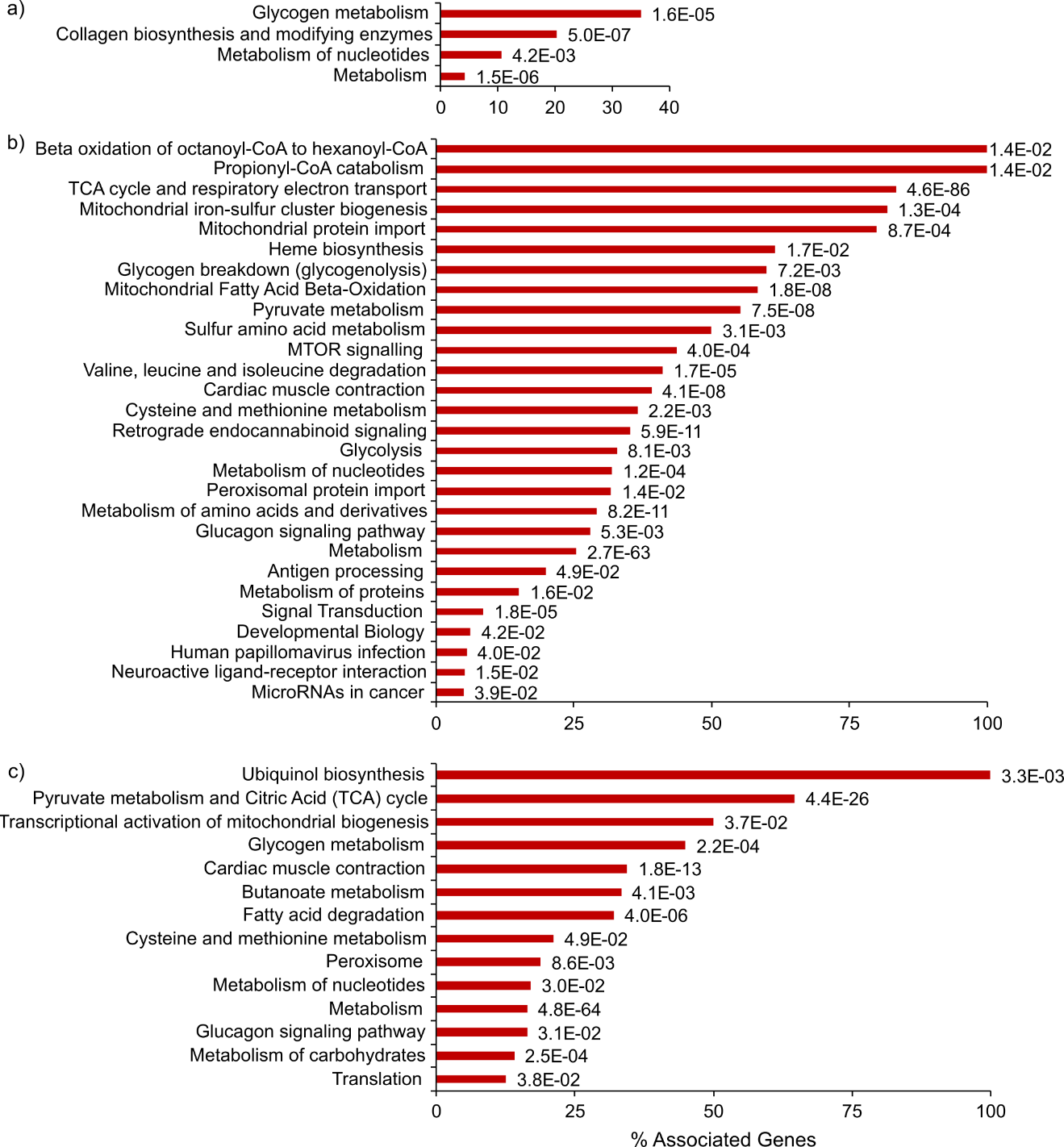
Figure S5: An verview of pathways enriched from the downregulated genes identified from *mdm* and wild-type comparisons in a) EDL, b) psoas and c) soleus.** Each downregulated gene set was searched against KEGG, Wiki and Reactome pathway annotations using ClueGO tool in Cytoscape application under p-adj cutoff of 0.05. Results were summarized based on the gene overlap among terms, and the representative term for each group was selected based on the p-adj value. The percent associated genes denotes the percentage of differentially downregulated genes associated with representatative pathway as a fraction of total annotated genes. Adjusted p-values associated with the pathways are shown at the right side of the bars.

**
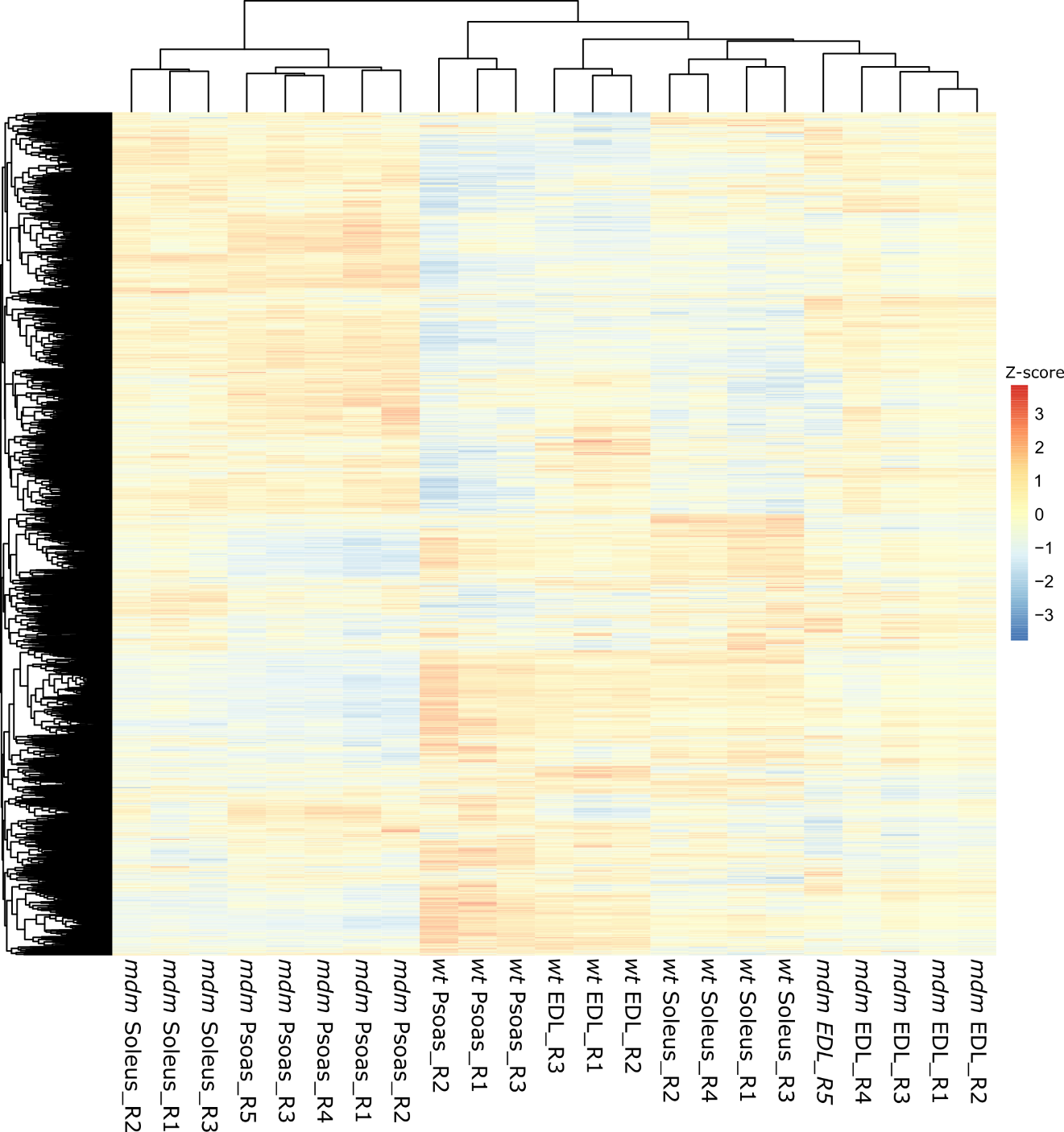
**

**Figure S6: Gene expression heat map of the complete data set, after removing marginally expressed genes.** The dataset was used for comparative analysis of *mdm­* muscles to wild-type muscles. Each row depicts a gene, each column depicts a muscle sample. Row and column clustering are based on the euclidean distance measures.

**
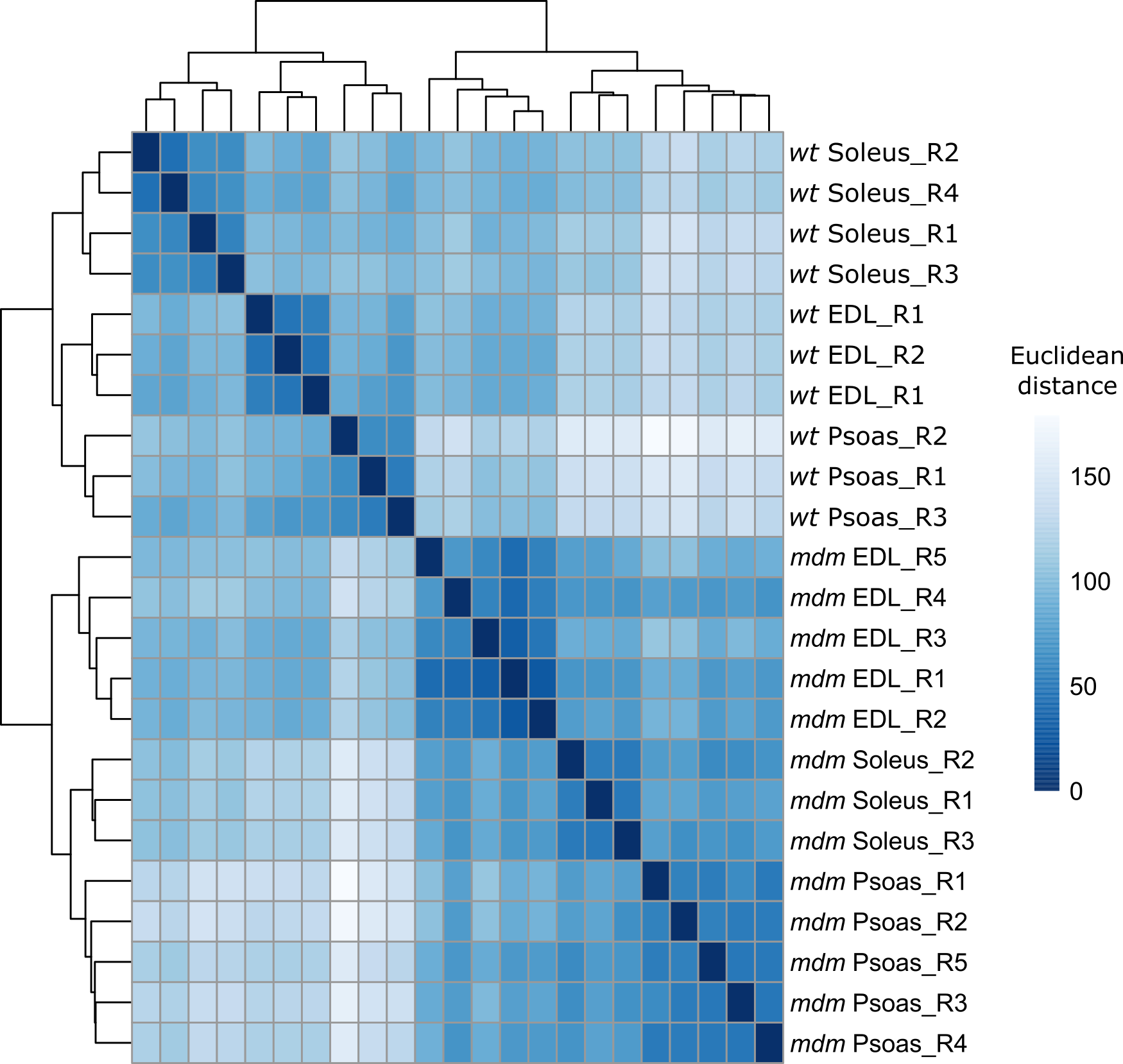
**

**Figure S7: Distance matrix among transcriptomic profiles show clear separation between *mdm* and wild-type samples**. Replicates of EDL, psoas and soleus muscles show clear clustering within the respective genotype. The distance matrix was calculated for the gene expression data set after removing marginally expressed genes

Table S1: Top 20 genes contributing to the variance represented by principal component l (PC1) and principal component 2 (PC2).

| 20 genes with highest loading to PC1 | Gene ID | Gene Description | Base Mean Expression | log_2_ fold expression change | | |
| --- | --- | --- | --- | --- | --- | --- |
|  |  |  |  | **EDL** | **Psoas** | **Soleus** |
|  | Dsp | Desmoplakin | 7708.43 | 10.51 | 10.06 | 8.04 |
|  | Myh3 | Myosin, heavy polypeptide 3, skeletal muscle, embryonic | 31030.03 | 5.88 | 7.00 | 6.37 |
|  | Mlf1 | Myeloid leukemia factor 1 | 3871.10 | -3.12 | -8.93 | -6.83 |
|  | Sln | Sarcolipin | 7097.70 | 8.62 | 8.96 | 3.94 |
|  | Krt18 | Keratin 18 | 1282.89 | 8.34 | 9.19 | 8.81 |
|  | Ankrd1 | Ankyrin repeat domain 1 (cardiac muscle) | 9631.11 | 4.24 | 8.53 | 5.11 |
|  | Cyp2e1 | Cytochrome P450, family 2, subfamily e, polypeptide 1 | 3294.63 | 5.57 | 5.69 | 5.60 |
|  | Scn4b | Sodium channel, type IV, beta | 5285.95 | -2.89 | -6.99 | -4.86 |
|  | Pvalb | Parvalbumin | 56924.28 | -3.15 | -7.32 | -3.18 |
|  | Ddit4l | DNA-damage-inducible transcript 4-like | 1701.90 | -3.59 | -6.84 | -5.57 |
|  | Krt8 | Keratin 8 | 469.85 | 9.38 | 9.22 | 11.76 |
|  | Pck1 | Phosphoenolpyruvate carboxykinase 1, cytosolic | 1598.89 | 5.20 | 5.47 | 5.12 |
|  | Cfd | Complement factor D (adipsin) | 9819.67 | 4.41 | 4.95 | 4.55 |
|  | Phkg1 | Phosphorylase kinase gamma 1 | 3738.65 | -2.62 | -6.09 | -4.02 |
|  | Tmem233 | Transmembrane protein 233 | 1076.31 |  | -7.31 | -5.45 |
|  | Myl4 | Myosin, light polypeptide 4 | 2020.49 | 3.48 | 6.29 | 3.89 |
|  | Rpl3l | Ribosomal protein L3-like | 3304.07 |  | -5.78 | -4.31 |
|  | Kcng4 | Potassium voltage-gated channel, subfamily G, member 4 | 591.86 |  | -8.15 | -6.76 |
|  | Igf2 | Insulin-like growth factor 2 | 2438.63 | 3.69 | 5.22 | 3.98 |
|  | Aldh3a1 | Aldehyde dehydrogenase family 3, subfamily A1 | 369.31 | 5.49 | 9.15 | 8.39 |
| 20 genes with highest loading to PC2 | Myh7 | Myosin, heavy polypeptide 7, cardiac muscle, beta | 71900.98 |  | 4.48 |  |
|  | Myl2 | Myosin, light polypeptide 2, regulatory, cardiac, slow | 28477.54 |  |  |  |
|  | Myl3 | Myosin, light polypeptide 3 | 15588.32 |  |  |  |
|  | Myh6 | Myosin, heavy polypeptide 6, cardiac muscle, alpha | 13204.66 |  | 3.18 |  |
|  | Tpm3 | Tropomyosin 3, gamma | 16718.74 |  | 2.83 |  |
|  | Tnnt1 | Troponin T1, skeletal, slow | 19173.41 | 3.14 | 5.46 |  |
|  | Tnnc1 | Troponin C, cardiac/slow skeletal | 2100.15 | 3.97 | 5.93 |  |
|  | Tnni1 | Troponin I, skeletal, slow 1 | 12844.57 | 4.06 | 6.99 |  |
|  | Atp2a2 | Atpase, Ca++ transporting, cardiac muscle, slow twitch 2 | 38385.75 | 2.52 | 3.52 |  |
|  | Strit1 | Small transmembrane regulator of ion transport 1 | 245.17 |  |  | -3.40 |
|  | Tpm3-rs7 | Tropomyosin 3, related sequence 7 | 623.26 |  | 1.67 |  |
|  | Bdh1 | 3-hydroxybutyrate dehydrogenase, type 1 | 1035.36 |  | -3.68 | -4.62 |
|  | Pvalb | Parvalbumin | 56924.28 | -3.15 | -7.32 | -3.18 |
|  | Mybph | Myosin binding protein H | 699.32 |  |  | 5.17 |
|  | Myh4 | Myosin, heavy polypeptide 4, skeletal muscle | 471407.73 |  | -6.63 |  |
|  | Mybpc2 | Myosin binding protein C, fast type | 42494.56 |  | -4.49 | -2.36 |
|  | Mhrt | Myosin heavy chain associated RNA transcript | 213.64 |  |  |  |
|  | Actn3 | Actinin alpha 3 | 49205.76 |  | -3.07 |  |
|  | Mettl21c | methyltransferase like 21C | 1735.63 |  | -2.47 |  |
|  | Actc1 | actin, alpha, cardiac muscle 1 | 4662.99 |  |  |  |

Table S2: RNA-Seq read alignment summary for the wild-type samples used in the study.

|  | | EDL_R1 | EDL_R2 | EDL_R3 | Psoas_R1 | Psoas_R2 | Psoas_R3 | Soleus_R1 | Soleus_R2 | Soleus_R3 | Soleus_R4 |
| --- | --- | --- | --- | --- | --- | --- | --- | --- | --- | --- | --- |
| Age (days) | | 42 | 54 | 44 | 42 | 54 | 44 | 42 | 44 | 32 | 44 |
| RNA integrity number (RIN) | | 8.4 | 8.4 | 8.5 | 7.3 | 8.1 | 8 | 8.2 | 8.1 | 7.6 | 8.1 |
| Library size | | 274 | 264 | 278 | 256 | 271 | 277 | 258 | 295 | 265 | 276 |
| Left reads: | Initial number of reads | 87185432 | 57368915 | 15186094 | 1E+08 | 45937372 | 13847980 | 22022374 | 28236087 | 12437526 | 15940351 |
|  | Minimum per base quality score (10th percentile) | 14 | 14 | 27 | 14 | 14 | 32 | 32 | 32 | 32 | 32 |
|  | Percent filtered in (%) | 81.38 | 80.83 | N/A | 81.86 | 80.26 | N/A | N/A | N/A | N/A | N/A |
|  | Alignment input | 70950705 | 46369117 | 15186094 | 81953371 | 36870545 | 13847980 | 22022374 | 28236087 | 12437526 | 15940351 |
|  | Mapped | 55572350 | 44539661 | 14774183 | 62419592 | 28077507 | 13534841 | 17953627 | 27374091 | 12109861 | 15593067 |
|  | Multiple alignments | 18.5% | 19.1% | 9.4% | 18.7% | 15.9% | 9.9% | 15.2% | 9.4% | 12.9% | 9.4% |
|  | Percent mapped | 78.3% | 96.1% | 97.3% | 76.2% | 76.2% | 97.7% | 81.5% | 96.9% | 97.4% | 97.8% |
|  | | | | | | | | | | | |
| Right reads: | Initial number of reads | 87185432 | 57368915 | 15186094 | 1E+08 | 45937372 | 13847980 | 22022374 | 28236087 | 12437526 | 15940351 |
|  | Minimum per base quality score (10th percentile) | 14 | 14 | 27 | 14 | 14 | 27 | 27 | 27 | 27 | 27 |
|  | Percent filtered in (%) | 81.38 | 80.83 | N/A | 81.86 | 80.26 | N/A | N/A | N/A | N/A | N/A |
|  | Alignment input | 70950705 | 46369117 | 15186094 | 81953371 | 36870545 | 13847980 | 22022374 | 28236087 | 12437526 | 15940351 |
|  | Mapped | 56026587 | 44570183 | 14597762 | 62738333 | 28269426 | 13259768 | 17647947 | 26044316 | 11953398 | 15344925 |
|  | Multiple alignments | 18.4% | 19.2% | 9.4% | 18.7% | 8.2% | 9.9% | 15.1% | 9.3% | 12.8% | 9.4% |
|  | Percent mapped | 79.0% | 96.1% | 96.1% | 76.2% | 76.7% | 95.8% | 80.1% | 92.2% | 96.1% | 96.3% |

|  | | EDL_R1 | EDL_R2 | EDL_R3 | EDL_R4 | EDL_R5 | Psoas_R1 | Psoas_R2 | Psoas_R3 | Psoas_R4 | Psoas_R5 | Soleus_R1 | Soleus_R2 | Soleus_R3 |  |
| --- | --- | --- | --- | --- | --- | --- | --- | --- | --- | --- | --- | --- | --- | --- | --- |
|  |  |  |  |  |  |  |  |  |  |  |  |  |  |  |  |
| Age (days) | | 38 | 38 | 42 | 38 | 38 | 29 | 38 | 42 | 38 | 38 | 33 | 36 | 38 |  |
| RNA integrity number (RIN) | | 8.6 | 8 | 7.9 | 7.8 | 8.1 | 7.7 | 7.8 | 7.8 | 7 | 7.5 | 8.7 | 7.5 | 7.8 |  |
| Library size | | 262 | 323 | 265 | 262 | 262 | 258 | 287 | 277 | 262 | 262 | 290 | 279 | 262 |  |
| Left reads: | Initial number of reads | 53885039 | 34932333 | 15793758 | 19829205 | 15148894 | 42440352 | 26748973 | 18199625 | 17724225 | 21623821 | 41514793 | 23439543 | 11885279 |  |
|  | Minimum average per base quality score | 32 | 32 | 32 | 32 | 32 | 30 | 32 | 32 | 32 | 32 | 32 | 32 | 32 |  |
|  | Percent filtered in (%) | NA | NA | 77% | 88% | NA | 82% | NA | NA | NA | NA | NA | NA | 87% |  |
|  | Alignment input | 53885039 | 34932333 | 12148686 | 17394262 | 15148894 | 34823448 | 26748973 | 18199625 | 17724225 | 21623821 | 41514793 | 23439543 | 10386271 |  |
|  | Mapped | 49508738 | 33952263 | 11887172 | 16686560 | 14436007 | 32915940 | 25658557 | 17475681 | 17003352 | 20516046 | 39386741 | 22523933 | 9583779 |  |
|  | Multiple alignments | 16.60% | 8.50% | 9.50% | 32 | 32 | 9.00% | 7.80% | 6.80% | 32 | 32 | 9.70% | 7.50% | 30 |  |
|  | Percent mapped | 91.90% | 97.20% | 97.80% | 95.93% | 95.29% | 94.50% | 95.90% | 96.00% | 95.93% | 94.88% | 94.90% | 96.10% | 92.27% |  |
|  | | | | | | | | | | | |  |  |  |  |
| Right reads: | Initial number of reads | 53885039 | 34932333 | 15793758 | 19829205 | 15148894 | 42440352 | 26748973 | 18199625 | 17724225 | 21623821 | 41514793 | 23439543 | 11885279 |  |
|  | Minimum average per base quality score | 32 | 32 | 32 | 32 | 30 | 30 | 27 | 27 | 30 | 30 | 27 | 27 | 32 |  |
|  | Percent filtered in (%) | NA | NA | 77% | 88% | NA | 82% | NA | NA | NA | NA | NA | NA | 87.39% |  |
|  | Alignment input | 53885039 | 34932333 | 12148686 | 17394262 | 15148894 | 34823448 | 26748973 | 18199625 | 17724225 | 21623821 | 41514793 | 23439543 | 10386271 |  |
|  | Mapped | 48848914 | 32872092 | 11658223 | 16644030 | 13857045 | 32889036 | 24621394 | 17269780 | 16465048 | 20072739 | 38831363 | 22091290 | 9557932 |  |
|  | Multiple alignments | 16.60% | 8.40% | 9.40% |  |  | 8.40% | 7.80% | 6.80% |  |  | 9.70% | 7.50% |  |  |
|  | Percent mapped | 91.70% | 94.10% | 96.00% | 95.69% | 91.47% | 94.40% | 92.00% | 94.90% | 92.90% | 92.83% | 93.50% | 94.20% | 92% |  |

Table S3: Alignment statistics of *mdm* samples used in the study.

**Table S4: Full gene ontology of differentially expressed genes (excel file)**
